# Exploring Transformations in Caribbean Indigenous Social Networks through Visibility Studies: the Case of Late Pre-Colonial Landscapes in East-Guadeloupe (French West Indies)

**DOI:** 10.1007/s10816-017-9344-0

**Published:** 2017-07-25

**Authors:** Tom Brughmans, Maaike S. de Waal, Corinne L. Hofman, Ulrik Brandes

**Affiliations:** 1 Department of Computer and Information Science, University of Konstanz, Konstanz, Germany; 2 Faculty of Archaeology, Leiden University, Leiden, The Netherlands

**Keywords:** Caribbean archaeology, Visibility, Network science, Guadeloupe, GIS

## Abstract

This paper presents a study of the visual properties of natural and Amerindian cultural landscapes in late pre-colonial East-Guadeloupe and of how these visual properties affected social interactions. Through a review of descriptive and formal visibility studies in Caribbean archaeology, it reveals that the ability of visual properties to affect past human behaviour is frequently evoked but the more complex of these hypotheses are rarely studied formally. To explore such complex hypotheses, the current study applies a range of techniques: total viewsheds, cumulative viewsheds, visual neighbourhood configurations and visibility networks. Experiments were performed to explore the control of seascapes, the functioning of hypothetical smoke signalling networks, the correlation of these visual properties with stylistic similarities of material culture found at sites and the change of visual properties over time. The results of these experiments suggest that only few sites in Eastern Guadeloupe are located in areas that are particularly suitable to visually control possible sea routes for short- and long-distance exchange; that visual control over sea areas was not a factor of importance for the existence of micro-style areas; that during the early phase of the Late Ceramic Age networks per landmass are connected and dense and that they incorporate all sites, a structure that would allow hypothetical smoke signalling networks; and that the visual properties of locations of the late sites Morne Souffleur and Morne Cybèle-1 were not ideal for defensive purposes. These results led us to propose a multi-scalar hypothesis for how lines of sight between settlements in the Lesser Antilles could have structured past human behaviour: short-distance visibility networks represent the structuring of navigation and communication within landmasses, whereas the landmasses themselves served as focal points for regional navigation and interaction. We conclude by emphasising that since our archaeological theories about visual properties usually take a multi-scalar landscape perspective, there is a need for this perspective to be reflected in our formal visibility methods as is made possible by the methods used in this paper.

## Introduction

This paper aims to explore transformations of indigenous social networks in late pre-colonial East-Guadeloupe from the perspective of visibility: a study of the visual properties of natural and Amerindian cultural landscapes, and of how these visual properties affected social interactions. The distribution of raw materials, goods and ideas reveal that the pre-colonial Caribbean was highly interconnected and dynamic (Hofman et al. 2007; Hofman and Bright 2010; Hofman and Hoogland 2011; Rodríguez Ramos 2010). The intervisibility of most Caribbean islands is believed to have played a structuring role in establishing social relationships (Hofman et al. 2007). Moreover, archaeologists have argued that lines of sight between Amerindian communities could have acted as media for the flow of information, which would have encouraged interactions, mobility and cultural exchange, also influencing settlement location selection (*e.g.* Bright 2011; Callaghan 2008; Hofman et al. 2007; Reid et al. 2014; Reid and Torres 2014; Torres and Rodríguez Ramos 2008). The study of visibility patterns is therefore considered an approach to understand one aspect of the social networks that connected indigenous communities in the past. But how exactly did visual properties of the natural and cultural landscapes of East-Guadeloupe affect human behaviour in pre-colonial times? How did these structures enable or hinder interactions between communities? And how did visual properties and their roles in Amerindian cultural landscapes change over time?

Our ability to answer these questions is affected by our fragmented knowledge of changing settlement, mobility and land use patterns as revealed by archaeological and historical sources, and by changes in the natural environment that may have taken place in the past. Moreover, the review of descriptive and formal visibility studies in Caribbean archaeology presented in the next section reveals that (although well-established in landscape archaeology in general) until now a very limited range of visibility analysis methods has been applied to answer a restricted range of research questions of limited complexity, such as the use of binary and cumulative viewsheds to determine the sea area visible from individual settlement locations. These methods are unsuitable for answering more complex questions such as the possible existence of smoke signalling networks or the ability of settlements to be hidden from view whilst being located nearby good vantage points. Brughmans and Brandes (2017) and Brughmans, van Garderen, Brandes, & Gillings (Introducing visual neighbourhood configurations for studying visual properties of landscapes. Journal of Archaeological Science, *in preparation*) have therefore developed an innovative GIS and network science approach to enhance our ability to explore complex visibility hypotheses, specifically designed for archaeological research contexts with fragmented knowledge of past landscape use. This paper presents the use of this approach to test and evaluate the complex visibility hypotheses that archaeologists have used until now to explain cultural landscapes in East-Guadeloupe.

**Fig. 1:**
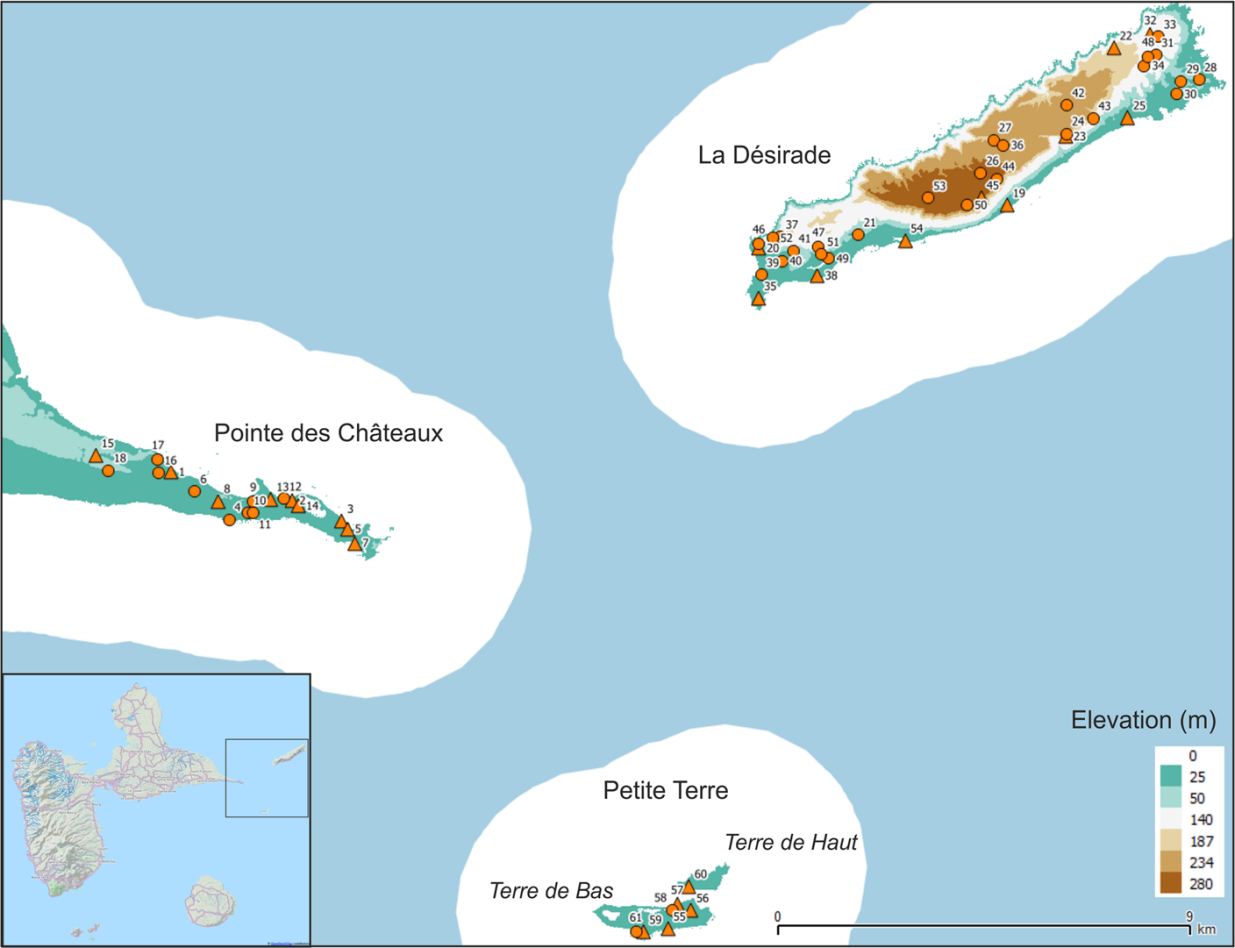
East-Guadeloupe study area with site locations used here (published in De Waal 2006; habitation sites represented as *triangles* and other sites as *circles*), listed in Table 1. *White area* represents 3 km buffer zones around landmasses in the research area, indicating maximum viewing distance from land used in this study. Elevation model Litto3D® Guadeloupe topographic LIDAR by © SHOM-IGN (2013). Inset © OpenStreetMap contributors

The present study focuses on East-Guadeloupe as for this region accurate pre-colonial site inventories are available due to systematic and intensive surveys and excavations (De Waal 1999, 2003, 2006; Hofman et al. 2001, 2014; Hofman and Hoogland 2011). The East-Guadeloupe region includes the Pointe des Châteaux peninsula of Guadeloupe and the islands of La Désirade and Petite Terre (Fig. 1). The period of interest concentrates on the early and late phases of the Late Ceramic Age (AD 600/850–1200/1300 and AD 1200/1300–1493, respectively), as important changes were observed between these phases with regards to settlement pattern, settlement structure, population density, site location choice and mobility and interaction (De Waal 2006; Hofman 1995; Hofman et al. 2004). We aim to explore in what way visual properties of the natural and cultural environment have influenced these changes (Table 1).

**Table 1:**
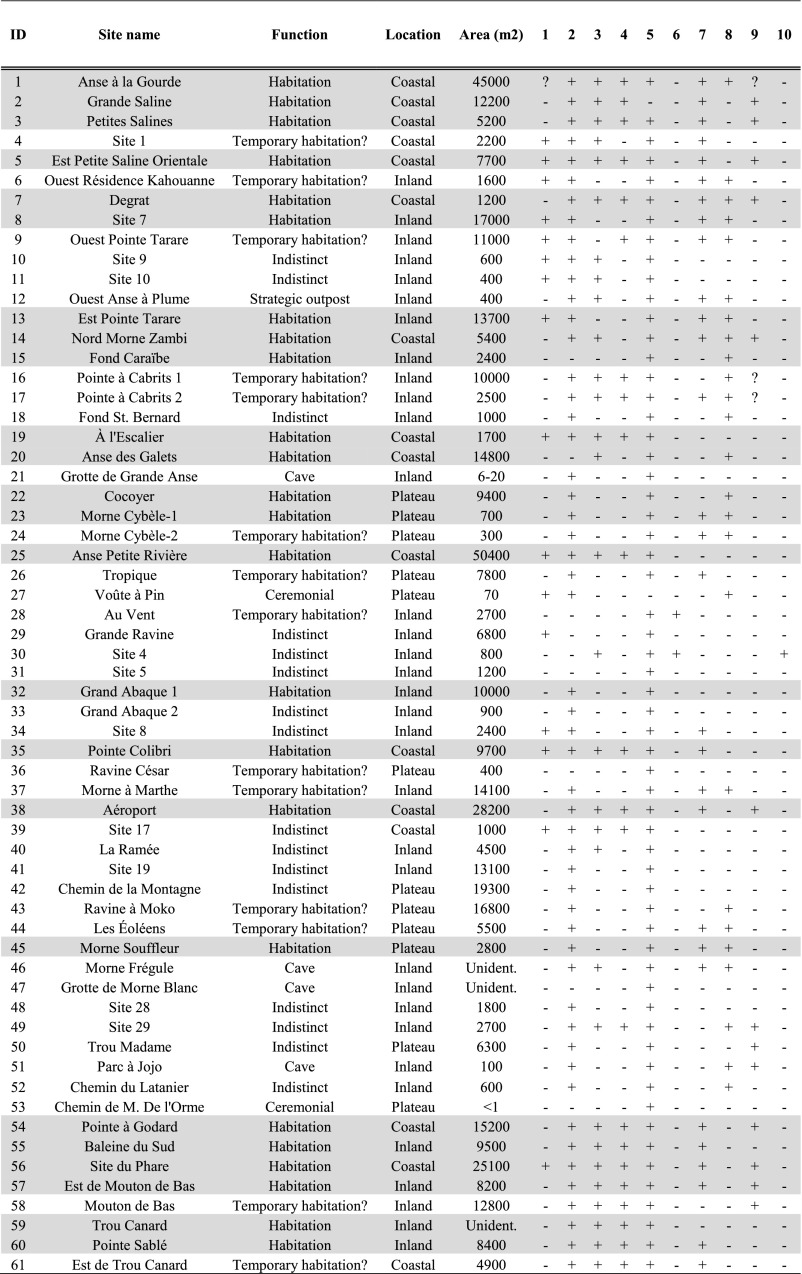
Late Ceramic Age sites in the East-Guadeloupe study area (published in De Waal 2006, 90–92). Sites believed to have a habitation function represented in grey

Site location variables record (sixth column onwards) presence of freshwater (1), flat terrain (2), accessible bays with canoe landing spots (3), reefs (4), soils suitable for small-scale horticulture (5), lithic raw materials (6), sites recorded as offering good viewpoints (7), strategic elevated spots (8), salinas (9) and mangrove (10)

Previous studies have emphasised the importance of intervisibility of different landmasses from site locations in East-Guadeloupe and the position of sites on opposing sides of islands, to understand the frequency of contacts or of cultural exchange between Amerindian communities on different islands (Bright 2011; De Waal 2006; Hofman et al. 2004, 2007). The ability to visually control sea areas crossed for short- and long-distance exchange and the ability to identify settlement locations from the visibility of smoke columns have equally been discussed in explanations of artefact distributions and settlement patterns elsewhere in the Caribbean (*e.g.* De Ruiter 2012; Ulloa Hung and De Ruiter 2011). Finally, the desire to remain largely invisible combined with an ability to visually control the surrounding landscape and seascape from vantage points nearby has been proposed as a partial explanation of the prominent locations of settlements like Morne Cybèle-1 and Morne Souffleur (dated to the latest phase of the Late Ceramic Age, *i.e.* AD 1460–1480) on the edge of the highly elevated central plateau of La Désirade (Delpuech 1998; De Waal 2006; Hofman 1995; Hofman et al. 2004), and was also proposed for contemporary sites in the region (Crock and Petersen 1999; Curet et al. 2004; Hofman 1995; Hofman and Hoogland 2011; Hoogland and Hofman 1999). These visual properties will be explored using total and cumulative viewsheds, visual neighbourhood configurations and visibility network techniques. Doing so will enable us to evaluate the changing role of visibility for Amerindian communities over time and to explore aspects of transformations of Amerindian social networks in late pre-colonial East-Guadeloupe.

## Visibility Studies in Caribbean Archaeology: Current State of Affairs

The role of visibility in mobility, cultural exchange and communication between pre-colonial indigenous communities, as well as Amerindian settlement location choice, has frequently been mentioned in Caribbean archaeological literature and has been studied through a variety of approaches. In this section, we review the hypotheses Caribbean archaeologists formulate about how particular visual properties might have affected Amerindian behaviour and cultural landscapes, and if and which formal methods have been used to explore these hypotheses.

### Descriptive Visibility Studies in Caribbean Archaeology

A number of studies refer descriptively to visibility patterns and mention hypotheses about how these could have affected human behaviour. The most commonly described visibility patterns can be divided into two categories: patterns argued to explain mobility and navigation, and patterns argued to explain site location.

Visibility patterns linked to mobility and navigation are most commonly mentioned with reference to the islands of the Lesser Antilles, most of which are intervisible (*e.g.* Hofman et al. 2007, 244). In the introduction to the *Encyclopedia of Caribbean Archaeology* the authors argue that the intervisibility of Lesser Antillean islands might have encouraged migration (Reid et al. 2014), an argument that is central to many of the visibility studies in Caribbean archaeology (Bright 2011; Callaghan 2008; Cooper 2010; Hofman et al. 2007; Reid and Torres 2014; Torres and Rodríguez Ramos 2008; Watters and Rouse 1989). The possibility to see one island from another or from specific site locations is often mentioned in studies based on archaeological surveys (*e.g.* De Ruiter 2012; De Waal 2006). Such accounts relate to the usually implicit hypothesis that the ability to see an island in the distance might have encouraged mobility, migration and frequent interaction and cultural exchange by acting as a marker for maritime navigation (*cf.* Bérard et al. 2011).

A number of studies link specific visibility patterns to these hypotheses. Not only the intervisibility of islands from land is considered important, but also the ability to see islands from sea. Visible islands and rocky outcrops can be used as markers for navigation. More specifically, areas in the sea between neighbouring islands from where islands are visible are considered to affect navigation and encourage migration. These spaces of intersecting visibility are argued to be particularly crucial in cases where islands are not intervisible from the landmasses themselves but only from the sea (Bright 2011; Callaghan 2008; Torres and Rodríguez Ramos 2008). Another example is offered by communities facing each other across a sea channel (so-called passage areas) who were not only closer to one another than to communities at opposite ends of the same island, but the sides of islands facing each other would also be intervisible. This pattern is hypothesised to encourage frequency of interaction and cultural exchange between communities (Bright 2011; Rouse 1951, 1982; Torres and Rodríguez Ramos 2008; Watters and Rouse 1989).

The second category of visibility patterns are those linked to explanations of site locations. In his introduction to the archaeology of the Caribbean, Wilson (2007, 81) mentions (but does not explain) that locations with good visibility on the windward sides of higher islands were preferred for early Ceramic Age settlement (400/200 BC–AD 400/600), whilst the islands preferred by the first Archaic Age people (5000 BC–AD 100) were avoided. A number of studies suggest specific hypotheses of how visibility patterns might explain settlement location: settlement location might have been influenced by (1) the ability to see large areas of the surrounding landscape or seascape (Bright 2011; De Ruiter 2012; De Waal 2006; Hofman et al. 2004), (2) the ability to see marine or terrestrial resource sites (Bright 2011; Cooper 2010; Delpuech 1998; De Ruiter 2012; De Waal 2006; Hofman and Hoogland 2011; Hofman et al. 2004), (3) the ability to enable communication through visible signals like light or smoke (De Ruiter 2012), (4) the ability to see approaching enemies with an eye on defence (Bradford 2001; Bright 2011; Delpuech 1998; De Waal 2006; Hofman 1995; Hofman et al. 2004; Hofman et al. 2007), (5) the ability to project one’s power or to emphasise the importance of a site location (De Waal 2006: 98) and (6) the ability to observe celestial bodies (Bradford 2001). Examples of several of these hypotheses are offered by the presentation of the Late Ceramic settlement patterns in East-Guadeloupe (see below). The passage areas hypothesis mentioned above also fits in this category, where settlements could have been located not only with an eye on monitoring and enabling mobility and navigation but also to enable frequent interaction, communication and exchange with (visible) communities on islands opposite.

Finally, a number of authors have argued that post-depositional processes and sea level change could have significantly altered the visibility of and from sites, and that this should be taken into account in both descriptive and formal studies (Cooper and Boothroyd 2011; Hofman et al. 2004). Cooper and Boothroyd (2011) performed a formal study of how Caribbean landmasses have shrunk through 5 m of sea level rise in the last 6000 years. Such changes in the area above sea level, coastlines and coastal topography caused by coastal erosion, sea level change, hurricanes, vegetation change and other post-depositional processes must significantly alter visibility patterns and our ability to accurately identify past visibility patterns. Such formal evaluations of these effects need to be incorporated in formal visibility studies like those introduced in the next section.

### Formal Visibility Studies in Caribbean Archaeology

The use of formal visibility methods is well established in archaeology in general (Lake and Woodman 2003), landscape-wide studies for addressing complex hypotheses like those presented in this paper have recently become more common (Eve and Crema 2014; Gillings 2015; Llobera 2003; Paliou et al. 2011), and theoretical issues surrounding the formal treatment of visibility are widely discussed (Gillings and Wheatley 2001; Wheatley and Gillings 2000). In this section, we will discuss formal visibility studies in Caribbean archaeology in more detail.

First, Torres and Rodríguez Ramos (2008) argue that a study of interisland visibility patterns through formal visibility analysis in GIS might allow one to better understand interisland interactions and past conceptualisations of landscapes. The authors reason that such a perspective focusing on connectivity allows Caribbean archaeologists to steer away from notions of insularity and isolationism so common in the study of island archipelagos. Instead, a relational perspective is needed to incorporate the human navigation factor. In this perspective the water should be seen as a relational space that is a continuum of the terrestrial social landscapes, rather than a separating space. The authors hypothesise that interisland visibility was an important variable in people’s decisions to migrate from one island to another and that overlaps in lines of sight from land to sea helped canoeists to navigate. Moreover, the intervisibility of islands is attributed the potential to enhance cultural interaction, a hypothesis that is indebted to Rouse’s (1992) concept of passage areas: stronger cultural relationships exist between communities on opposing islands than between communities on different ends of the same island (see also Bérard 2013; Bright 2011; Cooper 2008). However, we believe the analysis of what is visible from land suggests navigational decision making happened on land, and it would therefore be highly interesting to complement Torres and Rodríguez Ramos’ results with analyses of what is visible from sea: their interesting concept of the water as a relational space could be most appropriately represented through total viewsheds from land as well as from sea (as we illustrate for the case of Guadeloupe below).

A similar study is that by Callaghan (2008), who aimed to explore why no Archaic Age sites were found on Jamaica whilst they are attested on Cuba, Hispaniola and Puerto Rico. Callaghan considers a number of environmental factors that could have discouraged settlement in Jamaica: navigational difficulties, hurricane activity, sea level changes and visibility. Callaghan calculates maximum theoretical sighting distances between Jamaica, Cuba and Hispaniola, concluding that the islands are not mutually visible but that there are large areas in the sea where Jamaica and Hispaniola or Jamaica and Cuba are visible. This observation is elaborated by stating that travellers would not need to be out of sight of land for long and that the shortest travel route between Cuba and Jamaica in particular moves through the area of mutual visibility. Importantly, these distances calculated by Callaghan are maximum theoretical sighting distances that can be significantly reduced due to atmospheric conditions.

Jago Cooper (2010) provides another formal approach to study interisland connectivity incorporating visibility in his study of models of mobility and exchange in pre-colonial Cuba. Visibility is hypothesised to be one indication for frequency of interactions between island communities and the possibility for communities to control resources. The location of sites on higher elevations in particular is argued to have enabled the intervisibility of sites and the control of resources. To illustrate this, Cooper creates a cumulative viewshed of six sites in the study area (the sum of visible areas from each of the six sites, see below) and concludes that these sites are all intervisible, that from all sites similar upland areas can be seen, and that “the agroalfarero sites from the study area and the wider region are linked by visual connections that link the offshore islands, the coast and the Cuban interior” (Cooper 2010: 129). Moreover, none of the interior sites have views of the sea, which is taken to suggest that inland settlements did not have direct access to marine resources but were dependent on a coastal distribution centre to obtain these. Cooper further performs a cluster analysis of the site locations and a cost surface analysis simulating travel routes and times between pairs of sites, allowing for both terrestrial and maritime travel. He argues that the results of these formal analyses indicate that “inter-island and marine environment interaction in the case study area was direct and regular” (Cooper 2010: 133). Cooper’s study presents results that reflect particularly interesting visual properties of a number of site locations, but it could be enhanced in one important way. The cumulative viewshed approach used does not allow one to evaluate whether these sites’ visual properties are particularly exceptional for the study area: do other locations in the study area where sites could have been share these visual properties, and do the known site locations have exceptional intervisibility and control of resources in comparison? This can be evaluated by performing total viewshed and visual neighbourhood configuration experiments, as presented in this paper.

Finally, in their study of settlement patterns in the north-western Dominican Republic, de Ruiter (2012) and Ulloa Hung and Ruiter (2011) aim to evaluate what role visibility could have played in the selection of site locations. They calculated the percentage of the study area that is visible from sites and the percentage of sites visible from other sites (which allowed them to create a network of intervisible sites). De Ruiter (2012: 98) noticed that “Chicoid sites tend to be located more in the vicinity of other Chicoid sites than Meillacoid sites”. In addition, she noticed that even though Late Ceramic Age (Chicoid) sites are generally located at higher altitudes than contemporary (Meillacoid) sites, they nevertheless have more restricted views, for example because they are surrounded by higher hills. However, the Chicoid hilltop sites often offer views on the most important marine resource extraction sites. “The differences between Meillacoid and Chicoid sites in visibility ranges and the amount of sites visible are an indication that visibility did play a role in site location, and that it was not a ‘side effect’ of ecological factors” (De Ruiter 2012, 99). De Ruiter mentions that good visibility could have been purposeful to enable communication and views of the coast or resources. De Ruiter explores the possibility of enabling communication between sites through a visual signalling network by representing the intervisibility of sites as a network. However, De Ruiter does not provide an in-depth analysis of this network. Her study could be enhanced by evaluating the ability of this network to function as an efficient signalling network and the role of individual sites in this network through visibility network methods (as applied to the case of East-Guadeloupe in this paper).

### Hypotheses and Methodological Challenges

This literature review revealed that Caribbean archaeologists have formulated a large number of hypotheses of how visibility patterns could have affected pre-colonial human behaviour:

- Mobility and navigation: Intervisibility of islands encouraged mobility; areas of intersecting visibility in the sea encouraged mobility and acted as markers for navigation; visible features in the sea acted as markers for maritime navigation.
- Interaction and communication: Intervisibility encouraged interaction; communities facing each other on different islands encouraged interaction, communication and cultural exchange; communication between settlements through visible signalling.
- Control and surveillance: Defensibility, visibility of approaches to site; visual control of land and sea areas; visual control of land and marine resources.

Although formal GIS methods for the study of many of these complex hypotheses are well established in landscape archaeology in general, their use is less common in Caribbean archaeology. We argue that this would be a worthwhile pursuit for at least two reasons. First, this may allow us to build arguments for the importance of visibility in indigenous social networks, mobility and settlement location on reproducible and formally comparable results. Second, our fragmented knowledge of interactions between indigenous communities and of Amerindian natural and cultural landscapes requires evaluations of how well-known site locations fit the hypotheses as compared to all other locations in a study area. In other words: are the visual properties at known site locations exceptional when compared to the rest of the landscape where villages could have been located but where no sites have been found? Such an approach requires methods for formal comparison. In the remainder of this paper we will illustrate how formal visibility analysis addressing complex visibility hypotheses can lead to a better understanding of transformations of indigenous social networks.

## Transformations of Indigenous Social Networks in Late Pre-Colonial East-Guadeloupe

This study focuses on the changing visual properties of Amerindian cultural landscapes in East-Guadeloupe (Fig. 1) to better understand the changing social networks connecting Amerindian communities. The archaeology of this part of Guadeloupe is particularly well studied thanks to the intensive excavations at the site of Anse à la Gourde (Delpuech et al. 1999; Hofman et al. 1999, 2001, 2014; Hofman and Hoogland 2011) and an exhaustive regional survey (De Waal 2006). Previous studies of the diachronic settlement patterns and artefact distributions reveal Late Ceramic Age transformations of interaction networks between communities within the study area, as well as of long-distance interaction networks in the Lesser Antilles (De Waal 2006; Hofman et al. 2004, 2007; Hofman and Hoogland 2011). The presence of non-local materials at sites and the occurrence of stylistic similarities in ceramics reveal that the sea separating the landmasses of East-Guadeloupe encouraged interaction and exchange between Amerindian communities on different islands, linking island communities in social networks that transformed throughout the Late Ceramic Age (Hofman et al. 2004, 2007; Hofman and Hoogland 2011; Knippenberg 2007).

A notable settlement pattern change occurred in East-Guadeloupe between the early (AD 600/800–1200/1300) and late (AD 1200/1300–1493) phases of the Late Ceramic Age (De Waal 2006, 2014; occupation dates were mainly derived through relative dating of ceramics). The coastal areas of the study area were particularly densely occupied during the early phase, which also saw a more intensive use of the surrounding landscapes as compared to previous periods, the settling of the Petite Terre islands and the possible development of a settlement hierarchy centred on a few larger settlements like Anse à la Gourde (De Waal 2006; Hofman and Hoogland 2011). Our knowledge of the settlement pattern of the later phase of the Late Ceramic Age just before European contact is entirely different: most previously settled coastal locations, including all on Petite Terre, were abandoned, resulting in very sparse occupation of the area (De Waal 2006; Hofman 1995).

### Early Phase (AD 600/850–1200/1300)

The material culture from Late Ceramic Age villages in East-Guadeloupe suggests that they were part of both local and regional contact networks that transformed throughout the period (Hofman et al. 2004). A number of villages have particularly early dates, *ca.* AD 700/800–1000 and could have theoretically (based on possible overlaps in period of use) been in contact (De Waal 2006, 121–122): Degrat and Grande Saline on Pointe des Châteaux, and Grand Abaque 1 and Pointe Colibri on La Désirade. Moreover, close stylistic similarity of the pottery from Grand Abaque 1 and Pointe Colibri suggests that their inhabitants maintained regular contacts. The pottery from Anse Petite Rivière on La Désirade and Anse à la Gourde and other sites on Grande-Terre (Pointe Helleux, Pointe Canot and Pointe de la Couronne Conchou) make up a second stylistic group of sites settled from AD 1000 onwards, argued to reflect regular contacts between Amerindian communities in La Désirade and Grande-Terre (De Waal 2006, 124; Hofman et al. 2004, 167). Moreover, Anse Petite Rivière and Anse à la Gourde have demonstrably contemporary components covering the entire Late Ceramic Age and their inhabitants probably interacted through a short-distance contact network (De Waal 2006; Hofman et al. 2007).

The non-local lithics recovered from Anse Petite Rivière and Anse à la Gourde reflect the existence of long-distance exchange networks and their participation in these networks. Lithic raw materials suitable for the manufacture of stone tools were available on La Désirade and were attested at sites on La Désirade itself like Anse Petite Rivière, as well as at sites on Pointe des Châteaux like Anse à la Gourde. Communities on Petite Terre and Pointe des Châteaux also used flint from Antigua and other non-local rocks, in addition to lithics from La Désirade (De Waal 2006; Knippenberg 2007). The site of Anse à la Gourde seems to have been particularly pivotal in long-distance contact networks, as evidenced through the presence of green chert from St. Martin, igneous rock from Basse-Terre and Montserrat, metamorphic rock from the Greater Antilles or the South American mainland, and sandstone from Barbados and the Grenadines (Knippenberg 2007; Hofman and Hoogland 2011). We need to keep in mind, however, that the exceptionally intensive research activities at this site can bias our interpretation of its significance during this early phase of the Late Ceramic Age.

During this period, the islands of Petite Terre become settled for the first time and begin to serve as an important marine resource extraction area. Besides permanent habitation sites, temporary habitation sites have been identified on Petite Terre. They were probably “used by inhabitants of villages on Pointe des Chateaux and La Désirade, who were attracted by rich marine resources” (De Waal 2006, 117).

### Late Phase (AD 1200/1300–1493)

The density of population and number of villages significantly decreased towards the end of the Late Ceramic Age, when only three settlements were occupied in East-Guadeloupe: Anse à la Gourde at Pointe des Châteaux, and Morne Cybèle-1 and Morne Souffleur at La Désirade. Anse à la Gourde continued a long occupation history and is not located in a particularly defensive position. Morne Cybèle-1 and Morne Souffleur (Bodu 1985; Delpuech 1998; De Waal 2006, 2014; Hofman 1995; Hofman and Hoogland 1994) are argued to have been located in defendable places selected for their ability to observe humans approaching, or possible spiritual or symbolic appeal given the visibility of the La Désirade plateau with its table mountain shape from Petite Terre and Pointe des Chateaux (De Waal 2003, 2006: 128; Hofman 1995; Hofman et al. 2004). Even though these settlements were located at a large distance from each other (2.5 km as the crow flies) and were not in competition for resources or settlement locations, their founders selected locations with no direct access to freshwater or marine resources, which required trips of at least half an hour down the steep plateau side to obtain water and marine resources. Although both sites are very close to the plateau edge which offers great vantage points, the locations themselves are not very visible from the surrounding plateau landscape (De Waal 2006). A preference for elevated defendable locations might have continued into the period after European contact, as suggested by ethnographic accounts. Dreyfus’ (1976) summary of the sources on the seventeenth century Amerindian settlements confirms this, stating that settlements would have preferentially been in elevated locations where approaches to the settlement could have been observed. This defensive nature of settlement location and the importance of being able to observe possible enemies are also mentioned in the ethnographic accounts of Breton (1978 [1647]), and suggest that possible social changes in Amerindian communities before European contact caused them to abandon the exposed beaches and to occupy more discrete and defendable elevated locations instead (Delpuech 1998, 316). Although this is not the case for Anse à la Gourde, this picture is confirmed by recent investigations of early colonial Cayo sites on the islands of Dominica, St. Vincent and Grenada (Hofman 2016; Hofman and Hoogland 2012).

The pottery assemblages of Morne Cybèle-1 and Morne Souffleur share great stylistic similarity as does a small component of the Anse à la Gourde assemblage (De Waal 2006; Hofman et al. 2001, 2004). The ceramics are similar to the local Lesser Antillean Suazoid and Cayo pottery and show affiliations to the South American mainland and the Greater Antilles (Hofman et al. 2004; Hofman and Hoogland 2012). However, the presence at Anse à la Gourde of pebbles, magmatic rock and red chert from La Désirade suggests the continued existence of a contact network in the micro-region or of direct raw material procurement trips by Anse à la Gourde inhabitants. Moreover, the presence of Antigua flint and St. Martin green chert at Anse à la Gourde and Morne Souffleur suggests that the inhabitants of these villages were still part of long-distance contact networks. Due to the lower population densities, individual settlements had to sustain such networks over larger areas when compared to earlier periods (De Waal 2006).

## Research Questions

The main research questions we will explore relate to what extent these transformations in settlement patterns, and interaction networks can be understood through a study of visual properties of sites and the East-Guadeloupe landscape as a whole. These questions focus on four key topics:

1. Control of seascapes: The identification of land areas particularly suitable to visually control possible sea routes for short- and long-distance exchange may answer the following questions: are villages located in such areas and are they part of micro-style areas, or are other uninhabited areas more suitable to visually control seascapes? In which land areas are smoke columns rising up from villages visible from the sea, and from which sea areas are such smoke columns visible? Are villages located in such land areas and are they part of micro-style areas, or are other uninhabited areas more visible from the sea? Although it is known that the positions of the sun, moon and stars also may have played a role in navigation, only smoke signals were used in the present analysis. To simplify our communication of theories and results and to clearly distinguish this set of research questions from the others addressed in this paper, we will consistently use the word ‘control’ to refer to the diversity of archaeological theories stating that the possibility of surveillance might have been important to past communities, and to describe the results of our experiments to study these theories.
2. Signalling networks: The study of the intervisibility of eventual smoke columns in Late Ceramic Age sites as a visual communication or signalling network between communities may answer questions such as the following: in which ways can lines-of-sight structure interactions between communities in East-Guadeloupe? Did possibilities for interaction through visual signalling transform over time (*i.e.* are there differences between sites belonging to the particular micro-style areas)?
3. Micro-style areas: The comparison of visual properties of the sites belonging to the two micro-style areas that had been identified by De Waal (2006: 121–124) for the early phase of the Late Ceramic Age (one including Pointe Colibri and Grand Abaque 1, the other consisting of Anse Petite Rivière and Anse à la Gourde) and of the later stylistically linked sites (Morne Cybèle-1 and Morne Souffleur) may answer questions such as the following: do micro-style areas correlate with visibility patterns? Do sites of the same micro-area share visibility of similar natural features, or are their visual properties complementary? Are they visible to people navigating the coast in canoes? Do their locations allow visual control of sea areas? Are smoke columns rising up from stylistically linked villages intervisible, and what positions do they occupy in a hypothetical visual smoke signalling network?
4. Late phase network transformations and defensive locations: The exploration of long- and short-distance contact networks of the late phase of the Late Ceramic Age, by analysing visual properties of the three remaining villages (Anse à la Gourde, Morne Cybèle-1 and Morne Souffleur) may shed light on the following questions: how does their ability to visually control seascapes differ in comparison to the earlier sites? Are Morne Cybèle-1 and Morne Souffleur located in areas that serve a possible defensive function? Are they close to areas from which approaches to the sites, the coast and the sea can be better visually controlled when compared to other uninhabited areas?

## Data and Methods

A complete description of the data and methods used is available in the Appendix. In the current section, we provide a summary and a discussion of the methodological challenges that motivated the selection of the methods used and experiments performed.

To explore and test our research questions as hypotheses is challenging. First, the settlement pattern at the end of the Late Ceramic Age consists of only three sites, for two of which the preservation has been heavily affected by post-depositional processes. Second, we can also not exclude the existence of other contemporary sites destroyed by such processes or unidentified in previous archaeological activities. Third, the assumption that visual properties were vital for choosing these specific observed settlement locations implies that other suitable but apparently unoccupied settlement locations do not share these properties in the same manner. Fourth, the hypothesis that visual properties of settlement locations may help explain the observed changes in settlement patterns, implies that these properties were dissimilar during the different phases of the Late Ceramic period. The survey data provided in De Waal (2006, 90–92) as well as a visibility survey Brughmans conducted in the study area in 2015 include a qualitative assessment of site locations with good view points, which will be compared to the computationally derived results as a qualitative reference. But such qualitative observations do not allow us to distinguish between particular visual properties and how exceptional the visual properties at site locations are as compared to those of the entire landscape.

This study will address these challenges by performing total viewshed experiments (Llobera 2003) designed to represent and explore hypotheses of different visual properties for all locations in the study area (rather than only for the site locations). Cumulative viewsheds (Wheatley 1995) will be used to explore visual properties of sites belonging to micro-style areas and the later phase Late Ceramic Age sites. Total viewsheds will in turn be explored using a number of visual neighbourhood configurations (Brughmans et al., *in preparation*) to study how sites are embedded within local areas of particularly high or low visibility, and within areas of low visibility with good vantage points. Network science techniques will be used to represent and explore the intervisibility of sites (Brughmans and Brandes 2017). To address the issue of uncertain settlement sizes, we will explore all visual properties within the assumed maximum site area (as estimated in De Waal 2006, 90–91, Table 5.1) rather than referring exclusively to the results of point locations of sites.

**Fig. 2:**
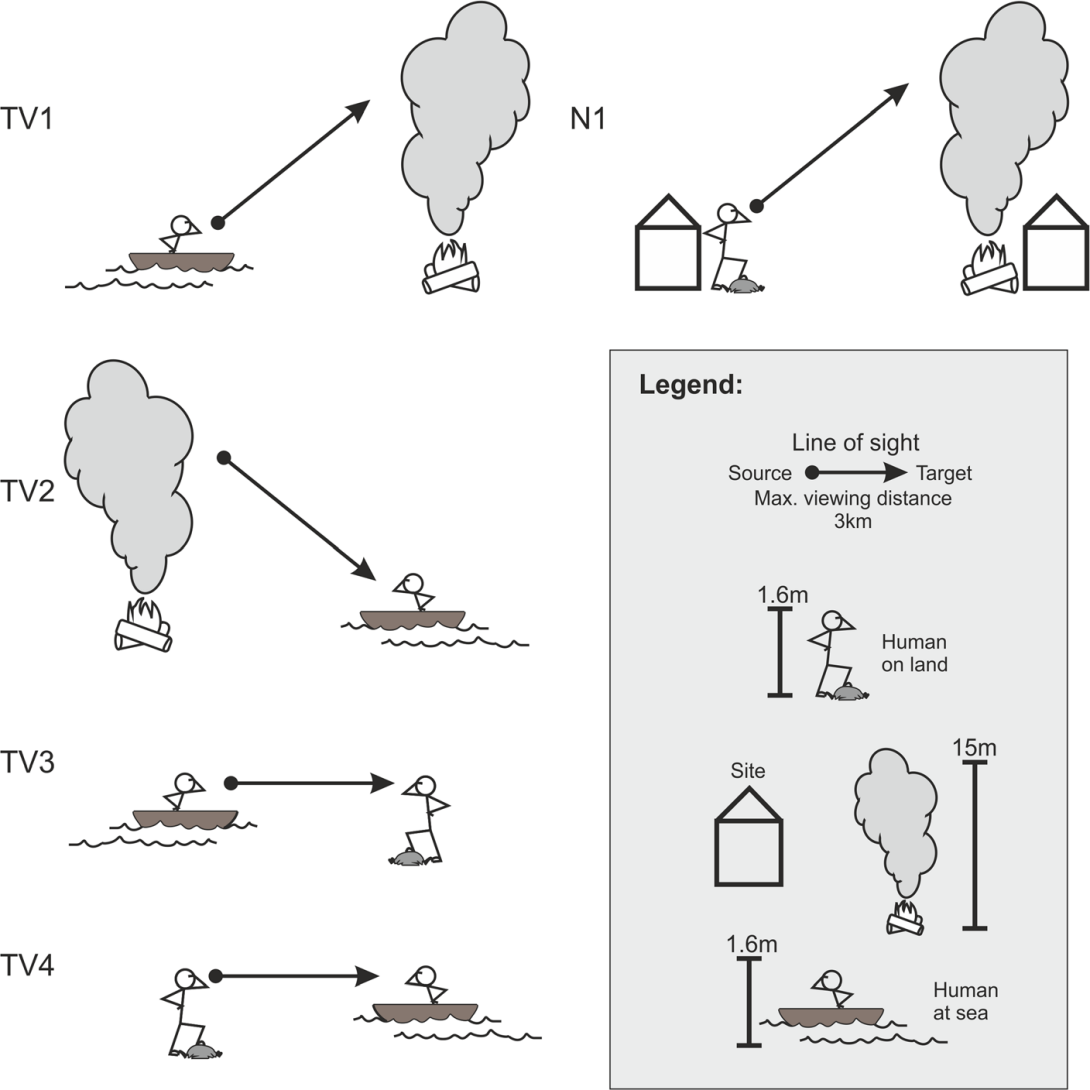
Abstract graphical representation of total viewshed (TV) and visibility network (N) experiments. In total viewshed experiments, values at target locations are recorded as results

All experiments performed and reported in the next section are listed in Table 2, and some are illustrated in Fig. 2. The motivation for experiment design, all variable settings, resolution of input data and software used is provided in full detail in the Appendix.

**Table 2:** Visibility experiments and variable settings

Total viewshed (TV) experiments	Source locations	Source elevation	Target locations	Target elevation	Max. distance	DEM resolution
TV1: land where smoke columns are visible from sea (views to smoke from sea)	Sea	1.6 m	Land	15 m	3 km	10 m
TV2: sea from which smoke columns can be seen (views from smoke to sea)	Land	15 m	Sea	1.6 m	3 km	10 m
TV3: land from which sea can be visually controlled (views to land from sea)	Sea	1.6 m	Land	1.6 m	3 km	10 m
TV4: sea that can be visually controlled from land (views to sea from land)	Land	1.6 m	Sea	1.6 m	3 km	10 m
Visual neighbourhood configurations (VNC) experiments				Input	Configuration	Area radius
VNC1: areas where smoke columns are on average highly visible from sea				TV1	Average	150 m
VNC2: locations where smoke columns are more visible than in their immediate surroundings				TV1	Prominence	150 m
VNC3: areas from which on average a large area of sea can be visually controlled				TV3	Average	150 m
VNC4: locations where sea can be better visually controlled than from their immediate surroundings				TV3	Prominence	150 m
Cumulative viewshed (CV) experiments	Source locations		Source elevation	Target elevation	Max. distance	DEM resolution
CV1: area where humans are visible from micro-style area 1 and contemporary sites	Pointe Colibri, Grand Abaque 1, Degrat, Grande Saline		1.6 m	1.6 m	3 km	5 m
CV2: area where smoke columns at micro-style area 1 and contemporary sites are visible	Pointe Colibri, Grand Abaque 1, Degrat, Grande Saline		15 m	1.6 m	3 km	5 m
CV3: area where humans are visible from micro-style area 2	Anse Petite Rivière, Anse à la Gourde		1.6 m	1.6 m	3 km	5 m
CV4: area where smoke columns at micro-style area 2 are visible	Anse Petite Rivière, Anse à la Gourde		15 m	1.6 m	3 km	5 m
CV5: area where humans are visible from late sites	Morne Souffleur, Morne Cybèle-1		1.6 m	1.6 m	3 km	5 m
CV6: area where smoke columns at late sites are visible	Morne Souffleur, Morne Cybèle-1		15 m	1.6 m	3 km	5 m
CV7: area visible from Anse à la Gourde	Anse à la Gourde		1.6 m	0 m	Unlimited	5 m
CV8: area from which Le Diamant is visible	Le Diamant		0 m	1.6 m	Unlimited	5 m
CV9: area visible from Anse Petite Rivière	Anse Petite Rivière		1.6 m	0 m	Unlimited	5 m
CV10: area visible from Morne Souffleur	Morne Souffleur		1.6 m	0 m	Unlimited	5 m
CV11: area visible from Morne Cybèle-1	Morne Cybèle-1		1.6 m	0 m	Unlimited	5 m
Visibility network (N) experiment	Source locations	Source elevation	Target locations	Target elevation	Max. distance	DEM resolution
N1: intervisibility of smoke columns at site areas	Site areas	1.6 m	Site areas	15 m	3 km	1 m

In total viewshed experiments, values at target locations are recorded as results. In cumulative viewshed experiments, the entire research area is the target location

## Results

For all total viewshed and visual neighbourhood configuration results, locations with results higher than one standard deviation added to the mean will be treated as locations from which ‘a larger area’ can be seen or that have ‘higher visibility’ (white in figures; bold in tables). Locations with results lower than one standard deviation subtracted from the mean will be treated as locations from which ‘a smaller area’ can be seen or that have ‘lower visibility’ (dark grey in figures; underlined in tables). Where sites are mentioned in this results section, their site numbers will be included in square brackets (see Table 1 for a full list of site names and numbers).

### Total Viewshed Experiments (TV)

**Fig. 3:**
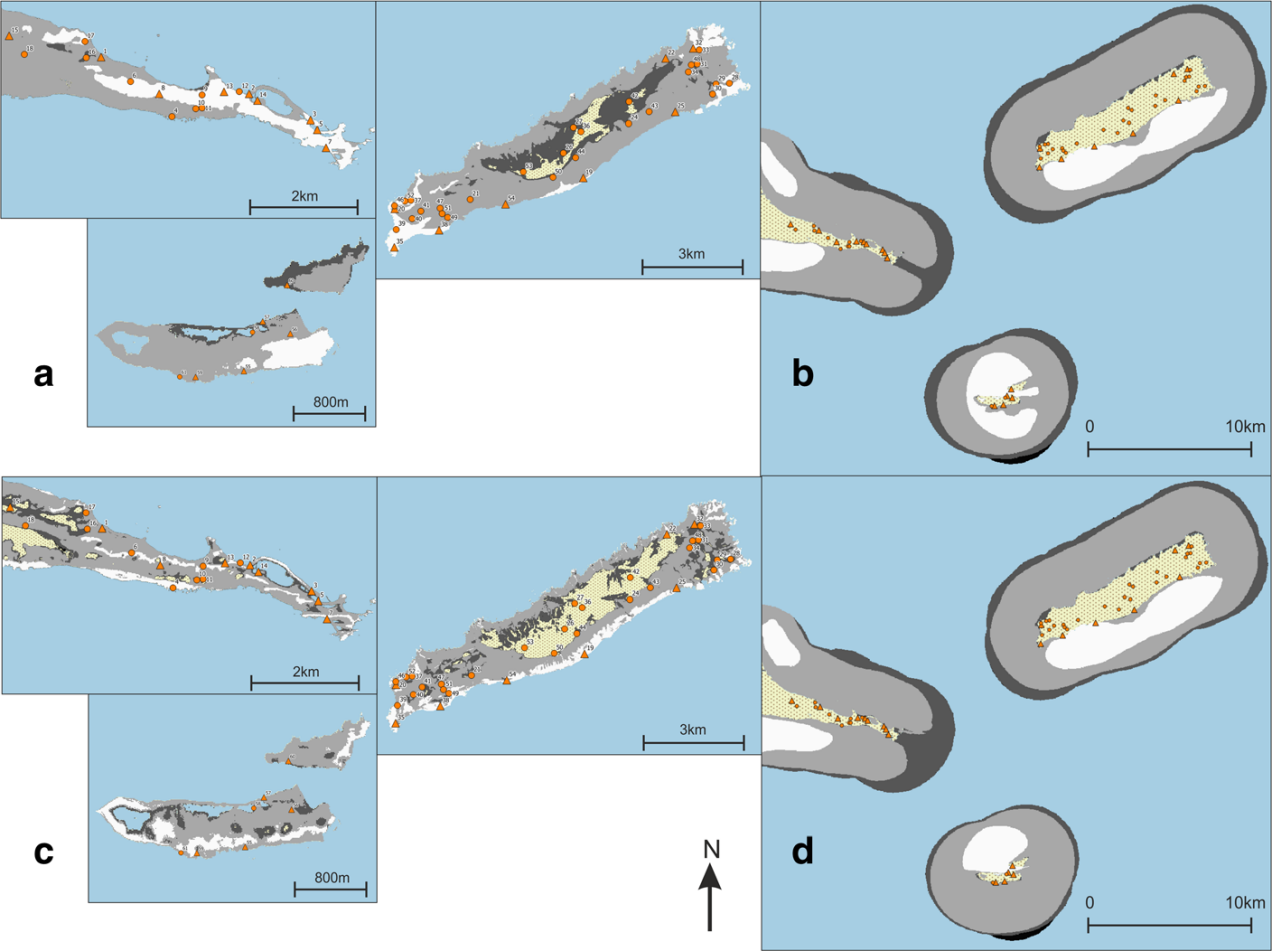
Results of experiments **a** TV1, **b** TV2, **c** TV3 and **d** TV4. Results higher than the mean plus one standard deviation represented in *white*; results lower than the mean minus one standard deviation represented in *dark grey*. Habitation sites represented as *triangles* and other sites as *circles*

Tables 3, 4 and 5 present per site the average result of experiments TV1 and TV3 within the site area; results of experiments TV1–4 are further shown in Fig. 3a–d.

**Table 3:**
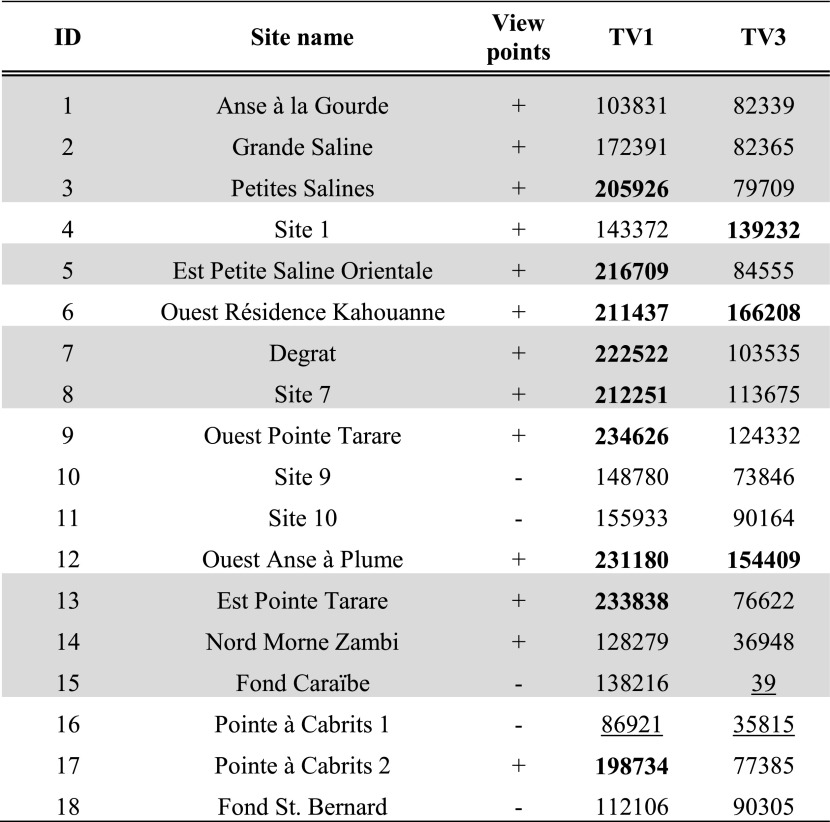
Average results for site areas on Pointe des Châteaux of experiments TV1 and TV3

Hypothesised habitation sites represented in grey. Results are classified in all subsequent tables as follows: results higher than the mean plus one standard deviation represented in bold, results lower than the mean and one standard deviation underlined. For easier comparison with experiment results, the ‘view points’ column of sites recorded through visits as offering good viewpoints (see Table 1) has been included in Tables 3, 4, 5, 6, 7 and 8

**Table 4:**
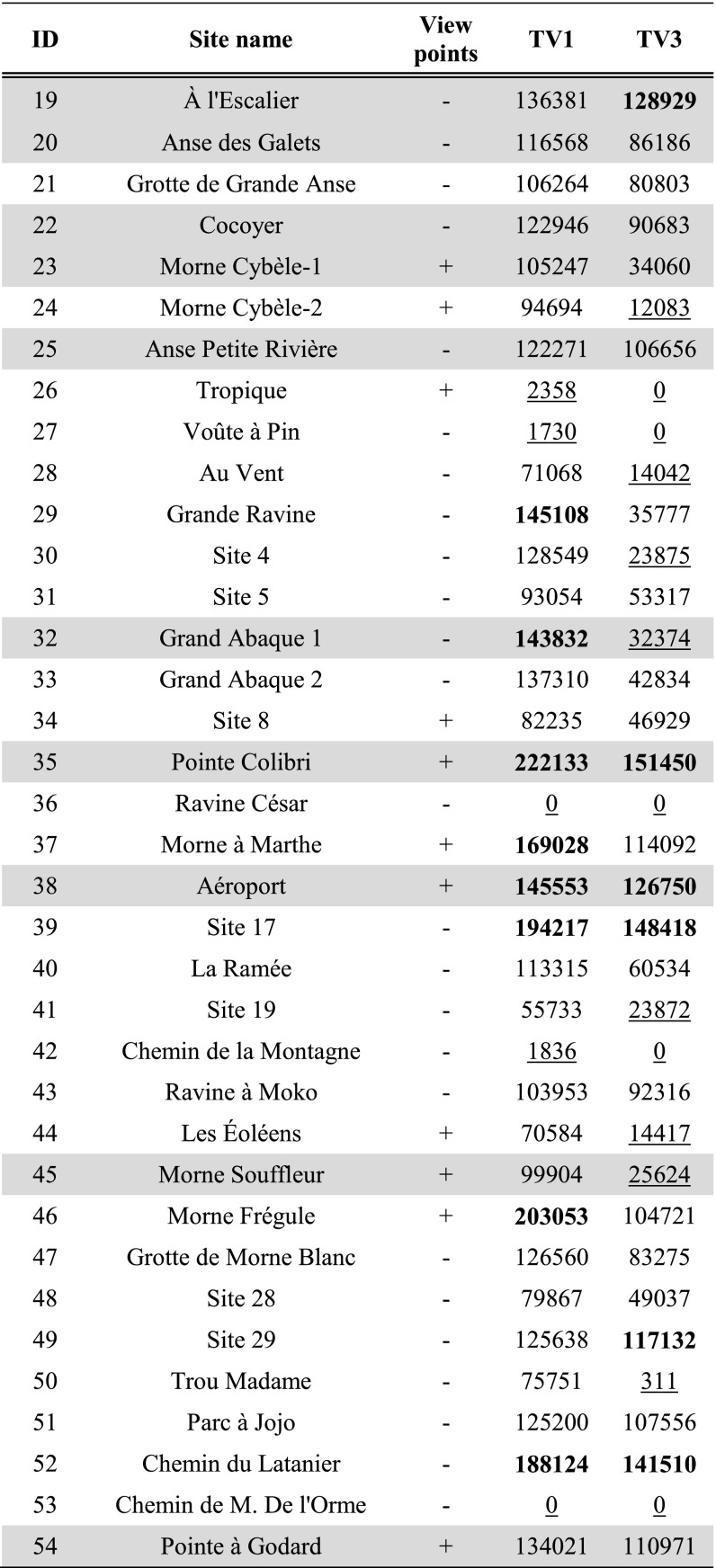
Average results for site areas on La Désirade of experiments TV1 and TV3

Hypothesised habitation sites represented in grey

**Table 5:**
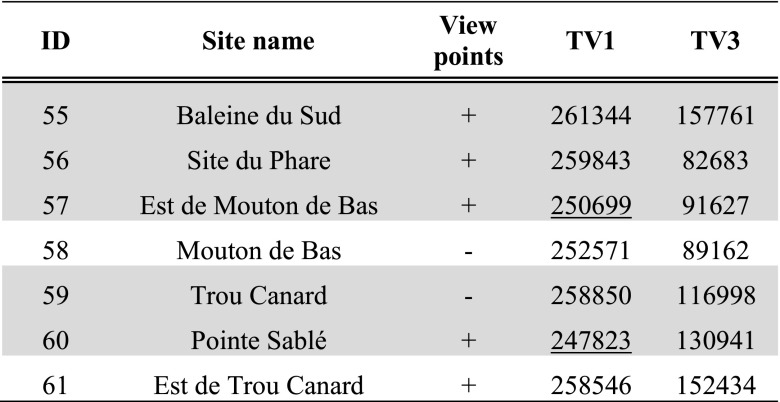
Average results for site areas on Petite Terre of experiments TV1 and TV3. Hypothesised habitation sites represented in grey

In the eastern part of *Pointe des Châteaux* (Table 3), including the area surrounding the salinas, smoke columns rising up from land would have been visible from large parts of the surrounding sea areas. These smoke columns could have been visible from sea areas both to the north and south of Pointe des Châteaux (TV1; Fig. 3a). Half of all sites are located in these areas of high visibility, whilst Pointe à Cabrits 1 [16] is the only site located in an area of low visibility from the sea. Habitation sites are located in areas of high visibility from the sea about as often as non-habitation sites. From sea areas along the southwestern and northwestern coasts of Pointe des Châteaux, smoke columns rising up from the largest land areas can be observed. This is important as a much larger land area lies within a 3-km radius of these western sea areas as compared to the sea areas in the east of Pointe des Châteaux (TV2; Fig. 3b). These same sea areas are also observable from the largest land areas (TV4; Fig. 3d). From parts of the eastern tip and a large part of the southern coast of Pointe des Châteaux, a large area of the sea leading to Petite Terre is visible (TV3; Fig. 3c). Interestingly, no habitation sites are located in areas of high visual control of the sea, whereas a number of non-habitation sites are located in such areas (site 1 [4], Ouest Résidence Kahouanne [6] and Ouest Anse à Plume [12]). The results of this experiment are more sensitive to the effects of vegetation and should therefore not be over-interpreted, although they do correspond to the qualitative assessment of sites with good view points (Table 1; De Waal 2006): all sites in areas of very high visibility and none of the sites in areas of very low visibility were qualitatively considered to offer good view points.

In both the western and eastern tips of *La Désirade* (Table 4), smoke columns rising up from land would be visible from large parts of the surrounding sea areas (TV1; Fig. 3a). Smoke columns rising up from the plateau would be visible from much smaller sea areas. Only eight sites are located in areas of high visibility, and interestingly, only three habitation sites are located in these areas, compared to five non-habitation sites (although all sites in areas of very low visibility from sea are non-habitation sites). It is important to note that the habitation sites in areas of high visibility include both sites belonging to the early micro-style area (Grand Abaque 1 [32] and Pointe Colibri [35]). From sea areas along the entire southern coast of La Désirade, smoke columns rising up from the largest land areas can be observed, whereas from many areas along the rocky and steep northern coast, very small land areas can be observed (TV2; Fig. 3b). These same sea areas are also observable from the largest land areas (TV4; Fig. 3d). From the western tip and the entire southern coastal area of La Désirade, a large area of the sea leading to Petite Terre is visible (TV3; Fig. 3c). Only three out of ten habitation sites are located in these areas of high visual control over the sea, including Pointe Colibri [35], one of the two sites of one of the early phase micro-style areas. Interestingly, the other site, Grand Abaque 1 [32], is located in an area of very low visual control over the sea. Three non-habitation sites are located in areas of high visual control of the sea. The results of this experiment are more sensitive to the effects of vegetation and should therefore not be over-interpreted, and they do not correspond well to the qualitative assessment of sites with good view points (Table 1; De Waal 2006): not all sites in areas of very high visibility and some sites in areas of very low visibility were qualitatively considered to offer good view points.

As for *Petite Terre* (Table 5), in the eastern part of Terre de Bas, smoke columns rising up from land would be visible from large parts of the surrounding sea areas (TV1; Fig. 3a). Smoke columns rising up from the northern coastal area of both islands of Petite Terre, facing La Désirade and Pointe des Châteaux would be visible from much smaller sea areas. No sites are located in areas that are highly visible from sea, whilst two habitation sites are located in areas of very low visibility (Est de Mouton de Bas [57] and Pointe Sablé [60]). From sea areas to the north-north-east facing Pointe des Châteaux, as well as sea areas to the south-south-west, smoke columns rising up from the largest land areas can be observed (TV2; Fig. 3b). However, the sea areas to the north-north-east facing Pointe des Châteaux are visible from particularly large land areas (TV4; Fig. 3d). The north coasts of Terre de Bas and Terre de Haut are not particularly good locations for observing a large sea area towards La Désirade and Pointe des Châteaux (TV3; Fig. 3c).

### Visual Neighbourhood Configurations Experiments (VNC)

**Fig. 4:**
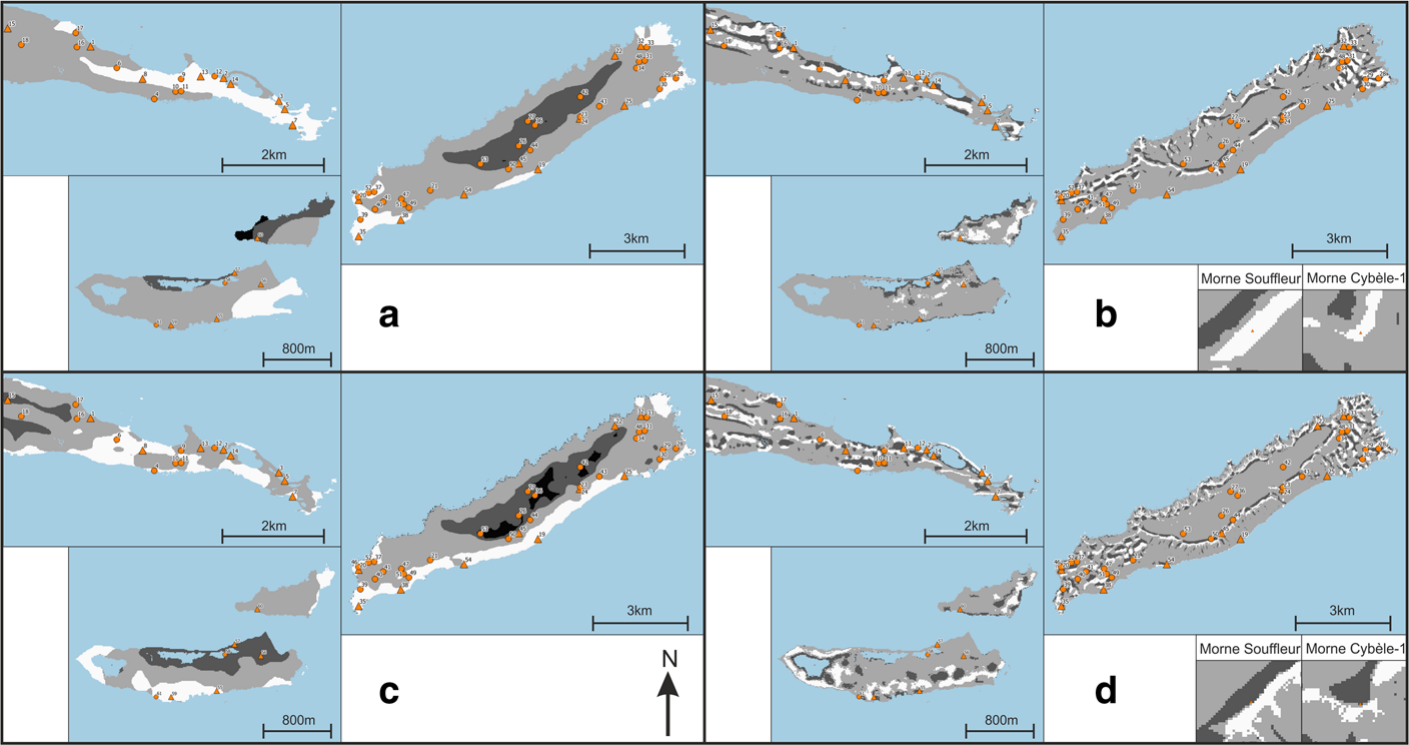
Results of experiments **a** VNC1, **b** VNC2, **c** VNC3 and **d** VNC4. Results higher than the mean plus one standard deviation represented in *white*; results lower than the mean minus one standard deviation represented in *dark grey*; results equal to zero represented in *black*. Habitation sites represented as *triangles* and other sites as *circles*

Tables 6, 7 and 8 present per site the average result of experiments VNC1–4 within the site area (Fig. 4a–d). Both sites belonging to the early micro-style area [32, 35] are located in areas where smoke signals on average are highly visible from sea (VNC1; Fig. 4a), and Pointe Colibri [35] is also located in an area of high visual control of the sea on average (VNC3; Fig. 4c). However, sites belonging to the other early phase micro-style area do not share any particular visual properties: Anse à la Gourde [1] does not have particularly high or low values for any of the VNC experiments, whereas Anse Petite Rivière [25] is located in an area of high visual control of the sea on average (VNC3; Fig. 4c). Morne Souffleur [45] and Morne Cybèle-1 [23], the two sites on the edge of the La Désirade plateau dated to the latest phase of the Late Ceramic Age, are located in areas where smoke columns are particularly visually prominent from sea, but very close to areas where they are not (VNC2; Fig. 4b; Table 7). These two sites are also located in areas from which much smaller sea areas can be visually controlled than from their immediate surroundings, but right on the edge of an area of very high visual control of the sea (VNC4; Fig. 4d; Table 7). This result supports the hypothesis that these two sites on the plateau edge are located in areas with high local variability in visual properties: large parts of the sites are located in areas with particularly limited visual control over the sea whereas small parts are located in areas offering great vantage points, whilst smoke columns at these sites would be visible from much larger sea areas than those in their immediate surroundings but close to areas where they would be far less visible. A number of other sites are similarly located in areas of high local variability of visual properties (Chemin du Latanier [52], site 17 [39], site 5 [31], site 4 [30], Au Vent [28], Morne Cybèle-2 [24], Anse des Galets [20], Cocoyer [22]).

**Table 6:**
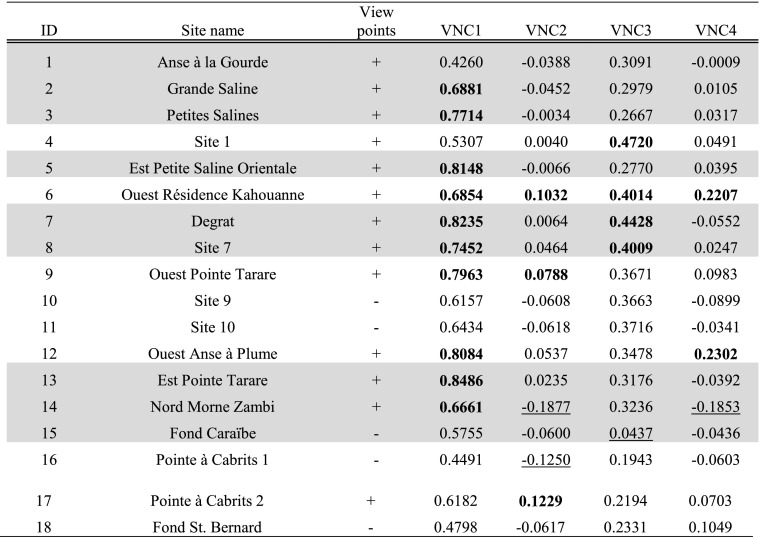
Results for sites on Pointe des Châteaux of experiments VNC1–4

Hypothesised habitation sites represented in grey

**Table 7:**
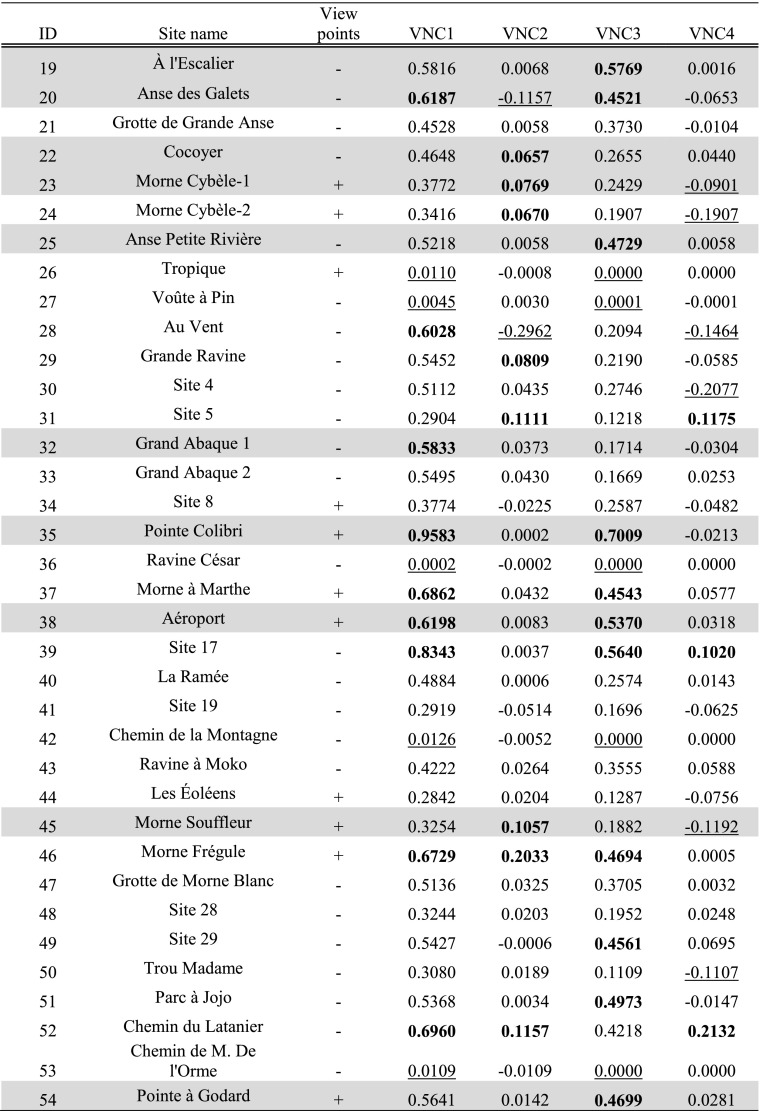
Results for sites on La Désirade of experiments VNC1–4

Hypothesised habitation sites represented in grey

**Table 8:**
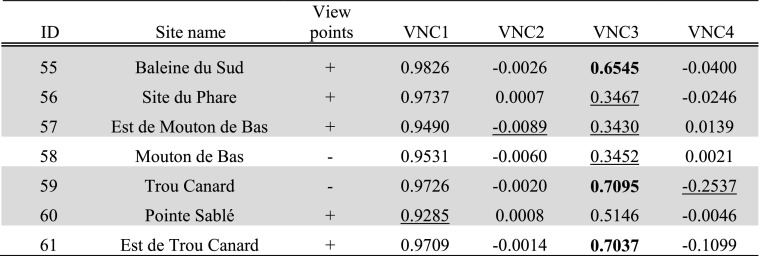
Results for sites on Petite Terre of experiments VNC1–4

Hypothesised habitation sites represented in grey

Most habitation sites on Pointe des Châteaux are located in areas where smoke columns on average are highly visible from sea, with the exception of Anse à la Gourde [1] and Fond Caraïbe [15] (VNC1; Fig. 4a; Table 6). However, no habitation sites on Pointe des Châteaux are in areas where smoke columns are highly visually prominent from sea (VNC2; Fig. 4b; Table 6). The site of Ouest Résidence Kahouanne [6] has a very high score for all four VNC experiments: it is highly visually prominent from sea, and from it a larger area of the sea can be visually controlled than from its immediate surroundings. A number of habitation sites on La Désirade are located in areas of high visual control on average over the sea: Anse Petite Rivière [25], Pointe Colibri [35], Aéroport [38], Pointe à Godard [54], À l’Escalier [19] and Anse des Galets [20] (VNC3; Fig. 4c; Table 7).

### Cumulative Viewshed Experiments (CV)

**Fig. 5:**
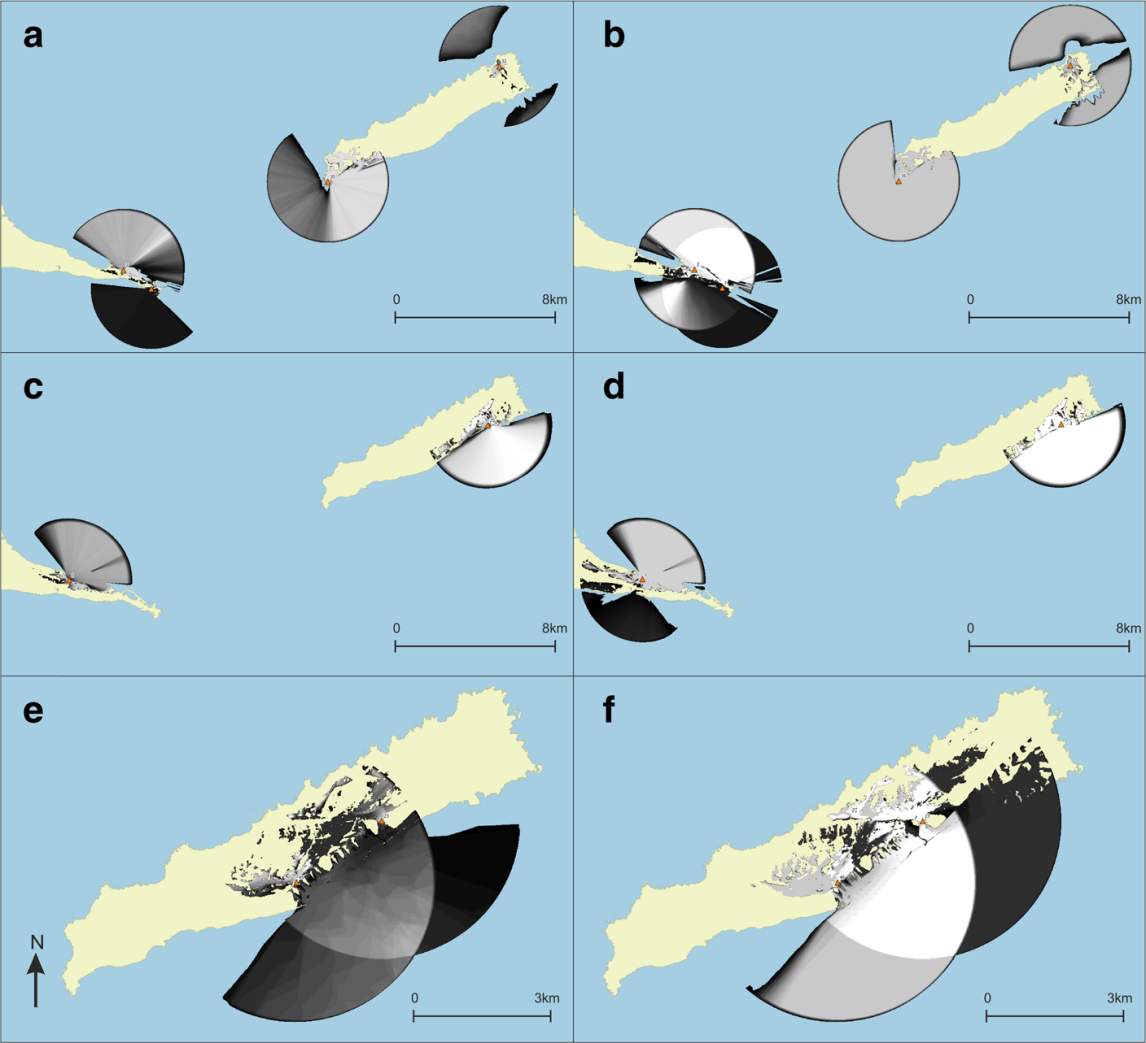
Results of experiments **a** CV1, **b** CV2, **c** CV3, **d** CV4, **e** CV5 and **f** CV6. Results represent number of observation locations within site area visible from a cell as a range from *black* to *white*: *black* = visible from 1 observation point, *white* = visible from maximum number of observation points in experiment

**Fig. 6:**
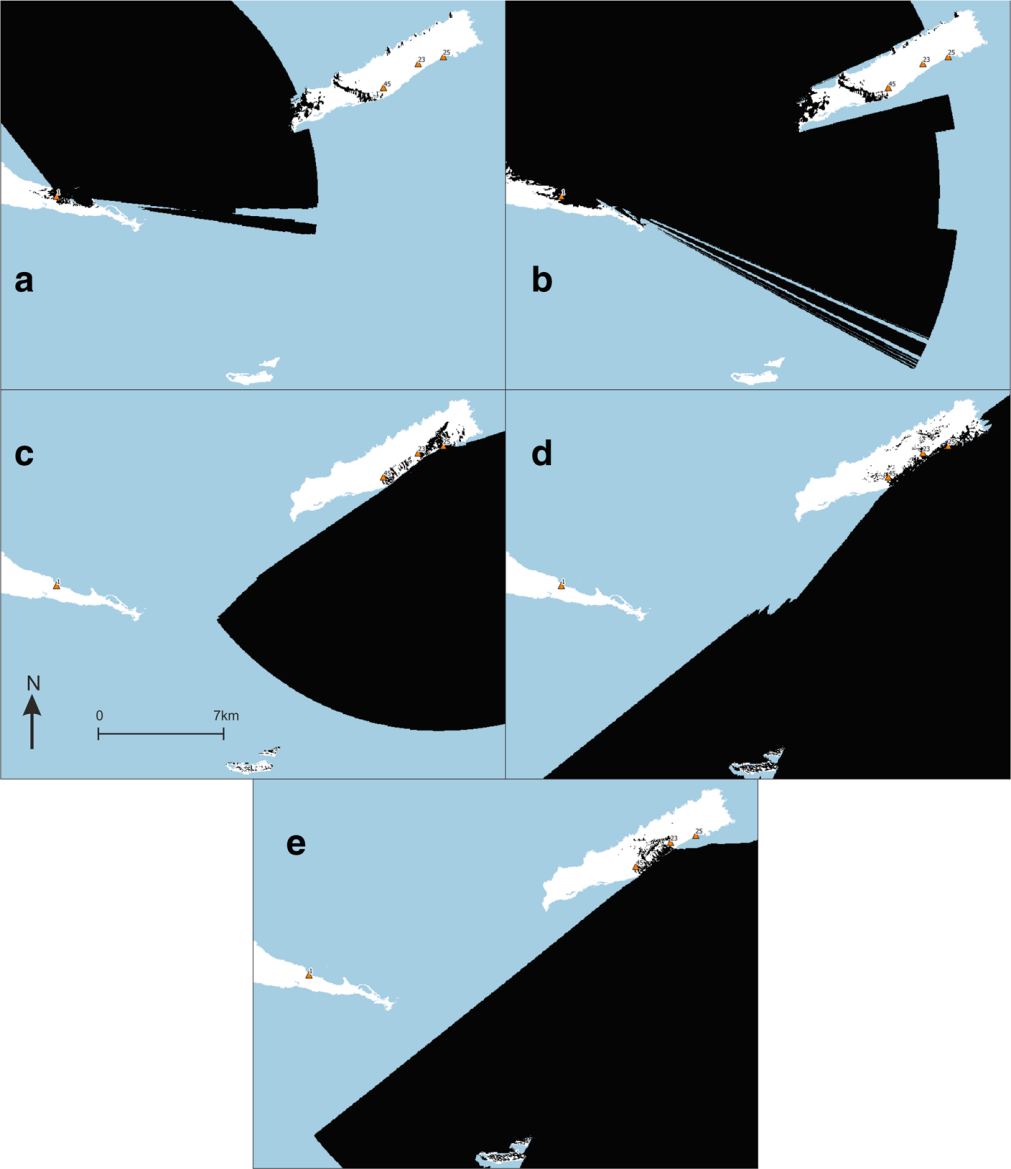
Results of experiments **a** CV7, **b** CV8, **c** CV9, **d** CV10 and **e** CV11. *Black area* is visible from at least one observation point in site areas

Few land and sea areas are visible from the sites belonging to the Pointe Colibri-Grand Abaque 1 [35, 32] micro-style area (CV1; Fig. 5a). The sites are spread out across La Désirade, and from both of them, different areas of the surrounding sea can be observed. However, smoke columns rising up from the contemporary sites Grande Saline [2] and Degrat [7] are both visible from large sea areas to the north and south of Pointe des Châteaux (CV2; Fig. 5b). The sites of the second early micro-style area (Anse à la Gourde [1] and Anse Petite Rivière [25]) are located far away from each other, are not intervisible and shared visibility of land or sea is impossible (CV3; Fig. 5c). From Anse à la Gourde [1] sea areas to the north can be observed as well as the western point of La Désirade (CV7; Fig. 6a) and smoke columns at the site are highly visible from this sea area (CV4; Fig. 5d). The rock off the coast of Anse à la Gourde [1], called Le Diamant, is visible from a very large sea area in between Pointe des Châteaux and La Désirade, as well as from the western point of La Désirade, and could have served as a marker for navigation (CV8; Fig. 6b). From Anse Petite Rivière [25] sea areas to the south can be observed and smoke columns at the site are highly visible from this sea area (CV3–4; Fig. 5c–d). The islands of Petite Terre can be seen from Anse Petite Riviere as well (CV9; Fig. 6c). Smoke columns from the two Late Ceramic phase sites on La Désirade (Morne Souffleur [45] and Morne Cybèle-1 [23]) are highly visible from a large land area on the central plateau, as well as from a large sea area along the south coast of La Désirade (CV6; Fig. 5f). This sea area can be visually controlled from parts of both sites’ areas, and much of the southern coast and plateau slopes can also be visually controlled (CV5; Fig. 5e). The islands of Petite Terre can be seen from Morne Cybèle-1 [23] and Morne Soufleur [45] (CV10–11; Fig. 6d, e). Pointe des Chateaux, however, cannot be seen from these two late sites, although during our visits to the sites we were able to observe the eastern point of this peninsula from vantage points at the very edge of the plateau near these sites.

### Visibility Networks (N)

**Fig. 7:**
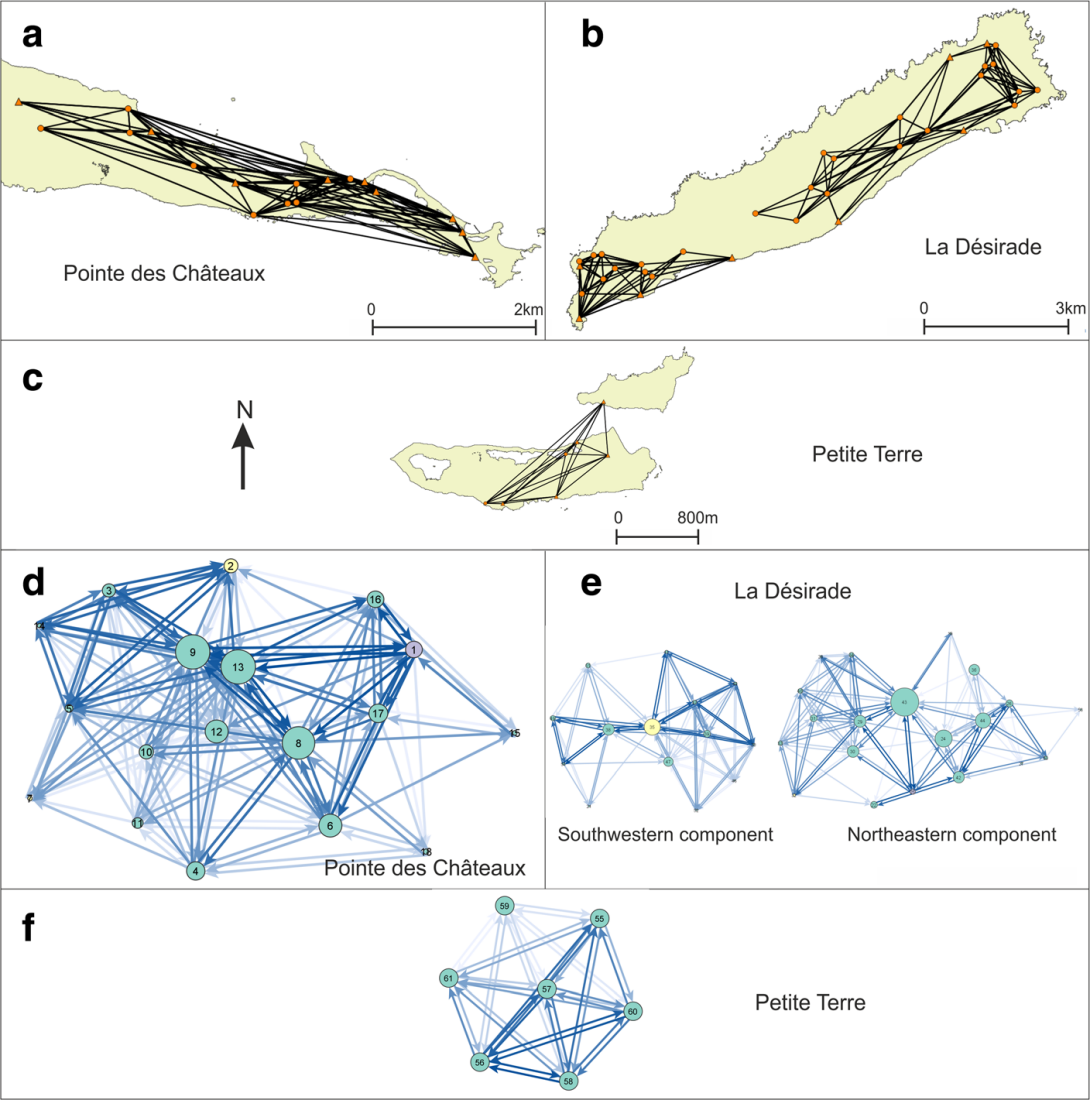
Visibility network N1 results represented as **a**–**c** geographical networks showing lines-of-sight present between site areas, and as **d**–**f** networks showing number of lines-of-sight between sites as line colour, betweenness centrality of sites as node size, and membership of micro-style areas as colour (*yellow* = Pointe Colibri and Grand Abaque 1, *purple* = Anse à la Gourde and Anse Petite Rivière). All sites are included in these networks. Habitation sites represented as *triangles* and other sites as *circles*

**Fig. 8:**
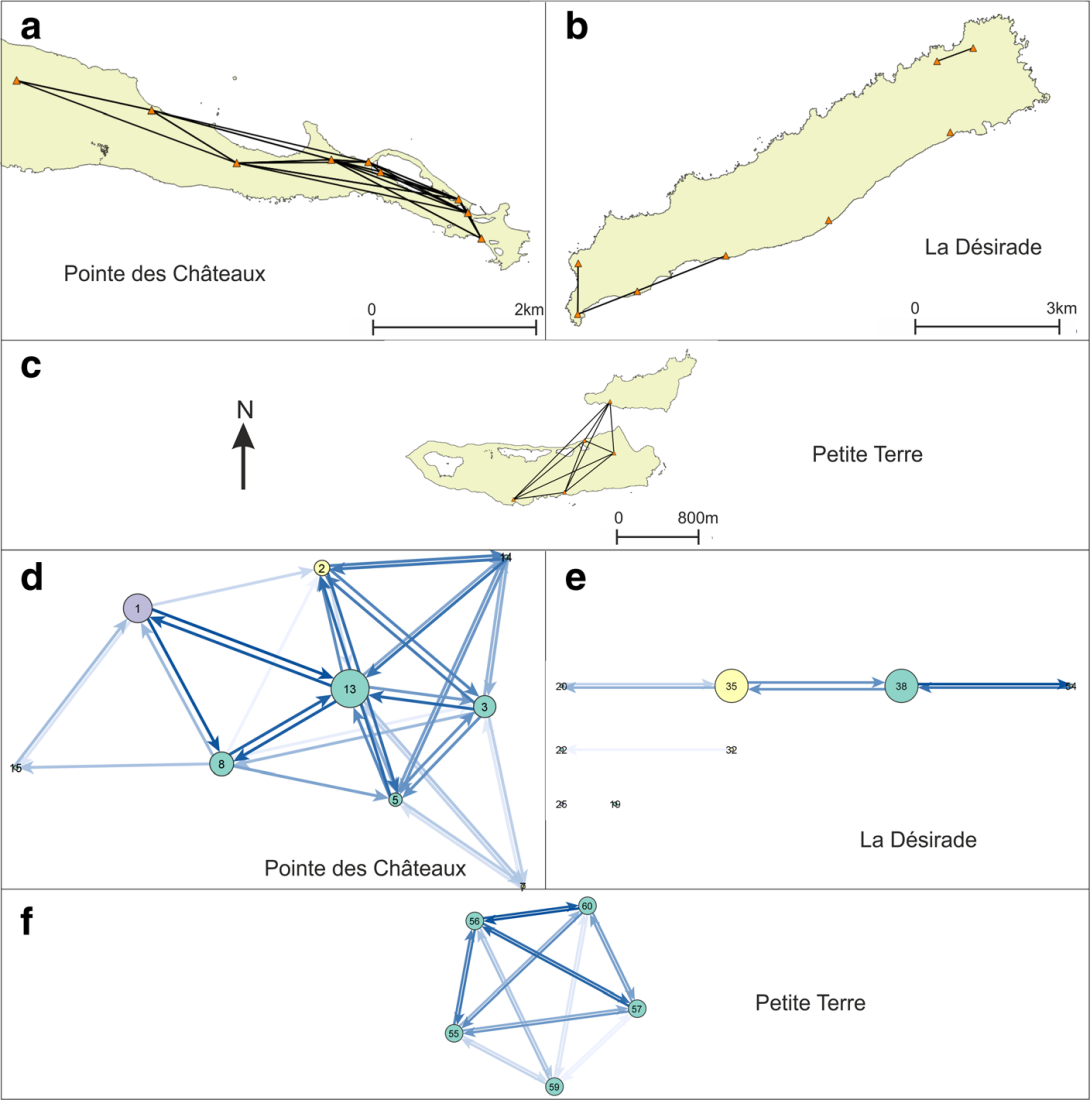
Visibility network N1 results, including only habitation sites, represented as **a**–**c** geographical networks showing lines-of-sight present between site areas, and as **d**–**f** networks showing number of lines-of-sight between sites as line colour, betweenness centrality of sites as node size, and membership of micro-style areas as colour (*yellow* = Pointe Colibri and Grand Abaque 1, *purple* = Anse à la Gourde and Anse Petite Rivière). Habitation sites represented as *triangles* and other sites as *circles*

The network of lines of sight between site areas is rather dense, all sites have at least one line-of-sight, and intervisibility (reciprocity) of smoke columns from site locations is high (Figs. 7 and 8). This result indicates that the physical environment of the research area would allow for the existence of a smoke signalling network between the known sites.

The visibility network on La Désirade consists of a south-western and a north-eastern component. The site of Pointe Colibri [35] (part of the early micro-style area) is rather highly visible: it has a high degree and connects two parts of the southwestern cluster, giving it a high betweenness centrality score. This suggests it would serve as an important intermediary in a hypothetical smoke signalling network. The weighted degree of this site in the area network is high, further confirming the site’s prominent role, but it is equalled by the very large site Aéroport [38].

Ravine à Moko [43], and to some extent Morne Cybèle-2 [24], play an important role as intermediaries between the eastern and the plateau sites in the north-eastern component. Grand Abaque 1 [32] has a very low betweenness score and is intervisible with a low number of other sites, whereas the weighted degree of Anse Petite Rivière [25] is very high due to a higher number of incoming and outgoing lines of sight from its large site area (these two sites are part of the two different early phase micro-style areas). From one site on La Désirade smoke columns at no other sites can be seen (Chemin de M. De L’Orme [53] on the central plateau).

Three sites at the centre of the Pointe des Châteaux research area are important as go-betweens (Ouest Pointe Tarare [9], Est Pointe Tarare [13], site 7 [8]). Anse à la Gourde [1] (part of an early phase micro-style area) has a high weighted degree but a low betweenness centrality. On Petite Terre, all sites are intervisible and therefore have equal betweenness scores.

When we remove the minor sites and only focus on the intervisibility of habitation sites, we notice an interesting pattern: villages on the south-western tip of La Désirade form an intervisible chain, which is mirrored in the intervisibility of villages on the north coast of Pointe des Châteaux facing this part of La Désirade across the sea (Fig. 8). In addition, Grand Abaque 1 [32] (early micro-style area) and Cocoyer [22] in the north-east of La Désirade are intervisible. On Pointe des Châteaux, the site of Est Pointe Tarare [13] has a high betweenness centrality score, whereas the part of Anse à la Gourde [1] that belongs to one of the early phase micro-style areas, has a high betweenness centrality and a particularly high weighted degree in the network with only habitation sites (Fig. 8).

Note that in Figs. 7 and 8 all Late Ceramic Age sites (excluding the sites of Morne Souffleur [45] and Morne Cybèle-1 [23]) are presented in the visibility network experiments, including the sites belonging to earlier and later micro-style areas, as eventual dating inaccuracies related to the use of relative dating based on pottery styles cannot be ruled out. However, experiments where we removed in turn sites belonging to either micro-style area revealed only minor changes to the network analysis results that do not affect the key results presented here. An exception to this is the removal of Pointe Colibri [35], which would break up the chain of intervisible habitation sites. Importantly, the removal of Anse à la Gourde [1] belonging to the other micro-style area does not break up the chain of intervisible habitation sites on Pointe des Châteaux facing the La Désirade chain. This suggests that the pattern of chains of intervisible habitation sites on opposing sides of landmasses is particularly robust during the period of occupation of Pointe Colibri [35] and might not have been so pronounced on La Désirade after this.

## Discussion

### Control of Seascapes

In this paper, we consistently use the word ‘control’ to refer to the collection of theories stating that the possibility of surveillance might have been important to past communities. We emphasise that results should be interpreted in light of this diversity of theories rather than exclusively the desire for exercising active control: the need to observe resources (like food or lithic extraction places) to avoid their use by certain people/communities; the ability to observe canoes navigating along the coast and individuals walking through the landscape to be aware of human movements, exchange routes or the possibility of attack. The outcomes of the experiments indicate the locations of land areas particularly suitable to visually control possible sea routes for short- and long-distance exchange. These include the southern coast and parts of the eastern tip of *Pointe des Châteaux* (TV3; Fig. 3c). These parts may have been particularly important in observing or controlling excursions between La Désirade and Basse-Terre. Only few sites are located in these areas. It is possible that visual control over both sides of Pointe des Châteaux could be exerted from other Grande Terre villages, located outside the study area (*cf.* De Waal 2006: Figs. 6.1, 6.3 and 6.8). In addition, as the Pointe des Châteaux peninsula only has a width of approximately 1 km, it is expected that communication between people in sites on the south coast with people in settlements in other parts of Pointe des Châteaux was quick and easy. Vantage points around major settlements like Anse à la Gourde could have enabled successful visual control over sea lanes to the north, east and south. The south coast is particularly well suited for visual control over the southern sea leading towards Petite Terre and enabling navigation further east to La Désirade (TV3; Fig. 3c). This is significant given the importance of Petite Terre as a marine resource extraction area (De Waal 2006: 65; De Waal 2009: 8–9) and of the hypothesised contacts between communities on Pointe des Châteaux and La Désirade. As sea currents to the north and north-east of Pointe des Châteaux are particularly strong (De Waal personal observations 1994–2002), we expect navigation from Guadeloupe to La Désirade to have followed the relatively sheltered southern coast before making the crossing to La Désirade. We therefore argue that sites on the southern coast should be considered lookout points as a complementary part of the major agglomeration of Anse à la Gourde on the north coast.

Interestingly, the temporary habitation sites of site 1 and Ouest Résidence Kahouanne, and the strategic outpost at Ouest Anse à Plume, are located in these areas controlling sea routes but permanent habitation sites are found in other locations (Table 3). This suggests that the location of Anse à la Gourde, one of the most prominent settlements in the study area thought to have played an important role in short- and long-distance exchange networks, was not selected for its visual control of sea routes. The Anse à la Gourde area has many other favourable conditions for a settlement (De Waal 2006: Table 5.1), but apparently, visual control of sea routes was not a requirement for a settlement to fulfil this role in exchange networks. Several uninhabited areas on the south coast were more suitable to visually control seascapes. It may also be suggested that other sites, such as site 1 and Ouest Résidence Kahouanne, fulfilled this role for Anse à la Gourde, even though they are at some distance (0.8 and 1.5 km) from this settlement. If this happened, this only occurred during the early phase of the Late Ceramic Age (AD 600/850–1200/1300). During earlier and later phases, Anse à la Gourde is surrounded by less sites, the landscape is more empty and the inhabitants of the settlement had fewer opportunities to control sea routes in their near surroundings.

Land areas particularly suitable to visually control possible sea routes for short- and long-distance exchange at *La Désirade* include the western tip and the entire southern coastal area of the island (TV3; Fig. 3c). It is noteworthy that the southern edge of the central plateau is not included. In the experiments, the plateau edge has normal visibility, but it has a lower theoretical maximum of visible sea area, being located slightly inland and given our theoretical maximum viewing distance of 3 km. When standing on the edge of the plateau at Morne Cybèle-1 and Morne Souffleur one can oversee the sea to Petite Terre (and identify the Petite Terre lighthouse) and to Pointe des Châteaux. From the plateau, it is also possible to see boats arriving at the island as landing areas for canoes can be observed. Morne Cybèle-2 overlooks Anse Petite Rivière and Morne Souffleur overlooks one of the other few bays where one can easily land a canoe at the south coast.

At La Désirade only six sites are located in areas with high visual control over sea routes (Table 4). Only three of these represent permanent habitation sites: Pointe Colibri, À L’Escalier and Aéroport. Grand Abaque 1, linked in a micro-style area with Pointe Colibri is in an area of low visibility. The natural setting of the sites of Pointe Colibri and Grand Abaque 1 is very different (De Waal 2006: Table 5.1). The regular contacts, suggested on the basis of similarities in ceramics, were apparently not linked to shared preferences for locations overlooking sea routes. They rather seem to complement each other, one site overlooking the west, the other site overlooking the east. Interestingly, habitation sites facing Petite Terre are located in areas of high visual control over the southern sea (Pointe Colibri, Aéroport, À l’Escalier TV3, Fig. 3c). Similarly to Anse à la Gourde, Anse Petite Rivière is located very close to (but not in) such areas (VNC3, Fig. 4c) (Table 4). Uninhabited areas close-by have slightly better visual control over seascapes. But no sites are close-by Anse Petite Rivière that may have taken over this function.

At *Petite Terre*, possible sea routes for short- and long-distance exchange can be controlled from large coastal areas except for the low-lying northern coast (TV3; Fig. 3c). No sites are located in areas with good visual control over seascapes (Table 5) and uninhabited areas allow visual control over seascapes. However, from Est de Mouton de Bas at the northeastern coast of Terre de Bas, and Pointe Sablé at the southern coast of Terre de Haut, one can control access to the restricted channel separating the islands of Petite Terre, which is reported to be one of the richest marine resource extraction areas for the islands (De Waal 2006: 65; De Waal 2009: 8–9). These sites may have played a role in mobility related to resource extraction at Petite Terre, for example by expeditions from La Désirade or Pointe des Château.

The results further indicate that in the eastern part of *Pointe des Châteaux*, including the area with the Salinas, smoke columns rising up from villages would be particularly visible from sea (TV1; Fig. 3a). Half of the sites are located in these areas, including half of the habitation sites, including Petites Salines, Est Petite Saline Orientale, Degrat and site 7 (Table 3). The sites are evenly distributed over these areas, which eliminates the question if uninhabited areas were more visible from the sea. Sites belonging to micro-style areas do not share the same characteristics related to the visibility of smoke columns. It is noteworthy that Anse à la Gourde is not in such a location. Regarding its role in regional and micro-regional networks one would expect that the visibility of smoke columns could help to direct interacting groups to the site. However, Anse à la Gourde does have an eye-catching feature that helps people navigating to the site: a small rocky island called ‘Le Diamant’, just north of Anse à la Gourde bay (CV8; Fig. 6b). In addition, even though visibility is low for the settlements of Anse à la Gourde and Grande Saline, high visibilities have been identified for the close-by indistinct site of Pointe a Cabrits 2, west of Anse à la Gourde, and of the strategic outpost at Ouest Anse à Plume, west of Grande Saline. These sites may have complemented this visibility role of both settlements. Smoke columns are best seen from sea stretches at the north-west and south-west of Pointe des Châteaux.

In the western and eastern parts of *La Désirade* smoke columns rising up from villages would be particularly visible from sea (TV1; Fig. 3a), especially from sea stretches directly south of the island. Only 8 out of 36 sites are located in these areas. Five of these are permanent settlements, including Pointe Colibri and Grand Abaque 1, which together form one of the early micro-style areas (Table 4). The latter site, which seems to be hidden in the eastern hills, must have been well visible when it comes to detecting smoke columns from the sea. However, it may be questioned how important the notion is that both early micro-style area sites have a similar visibility of smoke columns from the sea. Contacts between both sites may well have taken place over land, as they are only separated by approximately 10.5 km as the crow flies and as canoes cannot be landed at the northern and eastern sides of the island close to Grand Abaque 1. Interesting to note are the unremarkable results for the settlements of Morne Cybèle-1 and Morne Souffleur, neither particularly high or low. People establishing sites with presumably defensive locations probably tend to avoid attention to be drawn from a distance, but the results of TV1 (Table 4) do not provide strong support for this. Anse Petite Rivière also has a low visibility of smoke columns from the sea. Interesting to note as well is the high visibility of the south-eastern part of the island, where the (undated) lithic workshops are located. These workshops have played a role in micro-regional interactions as inhabitants from Pointe des Châteaux and from other parts of Guadeloupe collected raw materials for the manufacture of lithic artefacts at these locations (De Waal 2006: 106, 115, 130). The high visibility of the indistinct (special activity) site of Grande Ravine, at some elevation north of the lithic workshops, seems to complement the visibility of the latter.

In the south-eastern part of Terre de Bas in Petite Terre smoke columns rising up from villages would be particularly visible from sea (TV1; Fig. 3a). This seems important as this part of Terre de Bas is furthest removed from presumably important transportation routes between Marie-Galante (or further south) and the north, which may be expected to have passed the islands of Petite Terre in the west. No sites are located in these high visibility areas, whilst two habitation sites are located in areas of very low visibility (Table 5). Sea stretches surrounding the islands, except for the eastern part, would allow good views on smoke columns present on the islands of Petite Terre (TV2; Fig. 3b).

As a concluding remark, we cannot argue that the sites in general are located in places of exceptionally good or exceptionally bad visibility from and to the sea. Considering the presumed importance of the visibility of smoke columns with regards to intersite and interisland movements, more sites were expected to be located in high visibility areas than indicated in the experiments. The experiments do not indicate visibility to have been a factor of importance for the existence and functioning of micro-style areas either.

### Signalling Network

The visibility network experiment N1 (Figs. 7 and 8) revealed that the networks’ structures could have enabled smoke signalling networks between sites that could have functioned for communication purposes throughout the research area. The networks per landmass are connected and dense and they incorporate all known sites, allowing for information to be shared through signalling from any site to any other site.

The *Pointe des Châteaux* visibility network seems to consist of one single component. The settlement of Anse à la Gourde is a visually prominent node in this network. However, it did not play an important role as an intermediary. The *Petite Terre* visibility network includes one single component where all sites are intervisible (N1; Figs. 7 and 8).

**Fig. 9:**
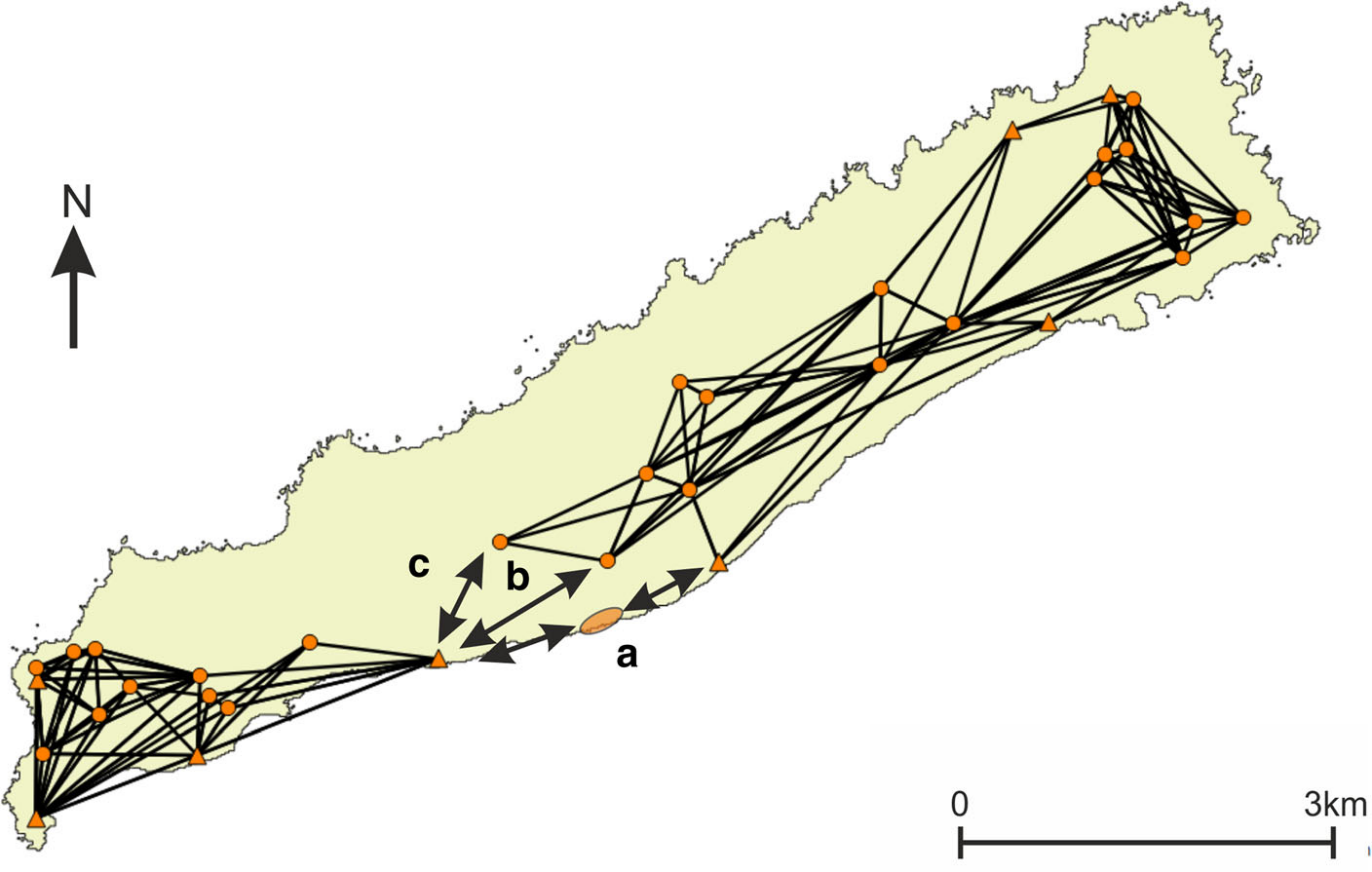
Hypotheses for connecting the two components on La Désirade (see N1; Fig. 7) to allow for an effective island-wide signalling network: **a** possible unidentified site along the coast, **b** good vantage points on the plateau edge nearby Trou Madame, **c** good vantage points on the plateau edge nearby Chemin de M. de l’Orme

The network on *La Désirade* consists of two separated components (N1; Fig. 7). Pointe Colibri plays an important role as an intermediary in a hypothetical signalling network, whereas Grand Abaque 1, in the eastern part of the island, does not play an important role. This network structure raises an important question: how could the western and eastern components become connected so that a functional signalling network could have existed for the entire island? There are multiple possible answers (Fig. 9). First, one is tempted to assume the existence of an unidentified coastal site between Pointe à Godard and À L’Escalier, but the coast is too steep here to allow settlement (Fig. 9a). Second, the site Trou Madame, tentatively interpreted as a (yet indistinct) special activity site, is located close to the plateau edge and within 3-km distance of Point Godard in the western component (Fig. 9b). Locations close-by Trou Madame offering lines-of-sight to Pointe à Godard would serve to connect both components. Third, and to us most intriguing, locations along the plateau edge nearby the site of Chemin de M. de l’Orme situated on the central plateau could also connect both components. Chemin de M. de l’Orme has been interpreted as a ceremonial, thus exceptional, site in an ‘empty’ area on the plateau (Fig. 9c). The site itself has low visibility (N1; Fig. 7) but nearby locations offer lines-of-sight to Pointe à Godard in the western component. It consists of a deliberate deposition of a small pelican vessel, which functioned as a container for a small non-used stone axe and adze of St. Martin chert (De Waal 2003, 2006: 99, 304). The site was considered a deposition in an unattractive area with no strategic importance, on a hilly terrain that is difficult to reach and far from the villages and the coast. In the light of the visibility analyses, it can now be suggested to have been close to locations that served as a lookout, connecting two network components on the island and it is tempting to further speculate if the depot was created as reference to this important role as a lookout or anchor point within the islands’ visibility network.

Interestingly, when we only include habitation sites, smoke signalling networks still may have functioned (Fig. 8). On the northern part of Pointe des Châteaux and the southern part of La Désirade, more or less ‘opposing sides’, the visibility networks of habitation sites form chains or paths through which information may have been shared from one side of a landmass to the other. This spatial distribution is thought not to be a result of the similarity in shape of the landmasses, as the pattern did not emerge in other similarly shaped parts of the study area. Due to the maximum viewing distance, artificially set at 3 km for our experiments, interaction between communities by smoke signalling was limited to intra-island contacts.

It is difficult to ascertain whether such hypothesised signalling networks transformed over time, since only a few sites could be assigned in an absolute way to different micro-style areas and phases of the Late Ceramic Age, making networks of securely dated contemporary sites impossible. However, one major change is obvious: a well-functioning smoke signalling network could have existed in the early phase of the Late Ceramic Age as it has a large number of sites all over the landscape, which allows intensive visual signalling networks, but not in the later phase. The decrease to only three later phase sites marks the end of a smoke signalling network if one did exist. Morne Souffleur and Morne Cybèle-1 are intervisible, but a non-trivial signalling network requires a chain of at least three sites where messages can be passed on between mutually intervisible sites A and C *via* a mutually visible site B. In addition, Morne Souffleur and Morne Cybèle-1 share similar visibility characteristics and do not complement each other’s observations. Interestingly, this later phase is precisely the period in which East-Guadeloupe inhabitants might have wished for relatively dense or tight signalling and communication networks in their close environs, as there would have been only few neighbours to rely on in times of need. Apparently, they depended on different ways to keep contact and they covered larger distances out of necessity (*cf.* De Waal 2006: 132).

The fact that most of the sites have not been absolutely dated reduces our certainty of these visibility networks’ structures. However, taking one node away from the network (for example because it is not contemporaneous with the other sites) does not cause the network to break up into multiple components, thanks to its density, and thus stop being able to function as a possible signalling network. When only habitation sites are included in the network the removal of a site has a larger impact, simply because less sites can take over its role. It is also interesting to note that when we look at the early phase of the Late Ceramic Age and in turn remove the sites belonging to each of the two micro-style areas, almost nothing changes in terms of the roles of other sites and the ability of the network to function. A problematic exception to this is the removal of Pointe Colibri, which would disrupt the ability of habitation sites on La Désirade to function as a visual signalling network.

### Multi-Scalar Networks

**Fig. 10:**
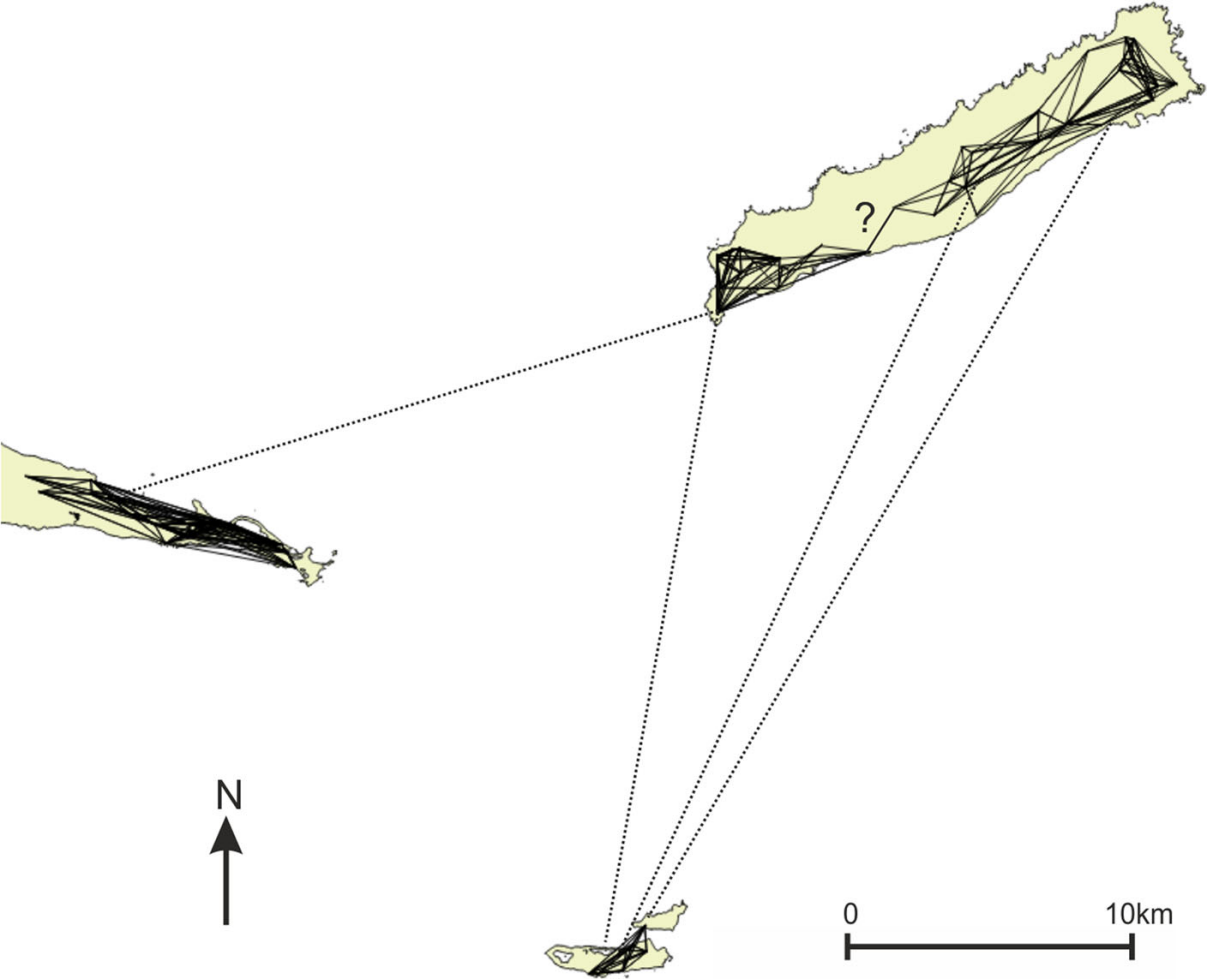
Representation of multi-scalar visibility network hypothesis: short-distance intervisibility of smoke columns create local networks on different landmasses (*solid lines*) that are connected into regional networks through long-distance intervisibility of landmasses (*dotted lines*). Hypothesised line-of-sight represented with *question mark*, see Fig. 9

We argue the significance of these visibility networks for structuring navigation and communication between communities in the area needs to be understood from a multi-scalar perspective: the chains of intervisible habitation sites created local (short-distance) networks on the different landmasses, and are linked up through long-distance visibility between the landmasses, where similar chains of intervisible sites mirror each other (Fig. 10). We hypothesise that the short-distance visibility networks structured navigation and communication within landmasses, whereas the landmasses themselves served as focal points for regional navigation and interaction.

The patterns in the study area support this hypothesis, but it may also serve as a hypothesis for the wider region. Habitation sites on the Pointe des Châteaux peninsula are regularly spaced and this pattern is mirrored along the western tip and southern coast of La Désirade, enabling a network of sites connected through the intervisibility of smoke columns (N1; Fig. 8). Outside the study area this pattern of regularly spaced habitation sites is continued along the northern and southern coast of Grande Terre (*cf.* De Waal 2006: Fig. 6.8), suggesting this visibility network could have been an important feature of the Late Ceramic Age settlement pattern of Grande Terre. These local chains are connected through the long distance intervisibility of landmasses from islands. Of particular importance is the visibility of the western side of La Désirade from Anse à la Gourde (CV7; Fig. 6a), and the visibility of Petite Terre and the eastern tip of Pointe des Chateaux from Morne Souffleur (De Waal and Hofman, personal observations 1994–2000). According to this hypothesis, direct visibility between contemporary sites might have been less important than the cumulative effect of a chain of long and short distance visibility: as soon as a new landmass was reached one could link into the local network of intervisible locations. For example, although the contemporary sites of Anse à La Gourde and Anse Petite Rivière are not directly intervisible, from Anse à la Gourde the landmass of La Désirade is visible (CV7; Fig. 6a), whereas the rock of Le Diamant off the coast of Anse à la Gourde is visible from a far bigger sea area south of La Désirade and might have served as an additional visual marker for people approaching this settlement from La Désirade and Anse Petite Rivière specifically (CV8; Fig. 6b).

This interpretation suggests that large parts of the landscape, vastly exceeding the boundaries of the East-Guadeloupe micro-region, were interconnected, even though the individual land masses are separated by long stretches of sea. These sea stretches could therefore be considered to have functioned as passage areas, linking the different land masses into local networks. This hypothesis further underlines the importance of understanding the visual properties of Late Ceramic Age East-Guadeloupe from a multi-scalar landscape perspective.

### Micro-Style Areas

The outcomes of the experiments indicate that sites belonging to the Pointe Colibri and Grand Abaque 1 micro-style area have similar visual property values. However, the sites belonging to the other early phase micro-style area, Anse Petite Rivière and Anse à la Gourde, have very different visual properties. With regards to visibility of people navigating the coast in canoes, Grand Abaque 1 and Pointe Colibri are located in areas where smoke columns are highly visible from the sea (TV1; Fig. 3a; VNC1; Fig. 4a), whereas Anse Petite Rivière and Anse à la Gourde, are located in areas with average visibility from the sea (TV1; Fig. 3; VNC1; Fig. 7). Visual control over sea areas does not seem to have been a factor of importance for the early phase micro-style areas. Pointe Colibri is one of only three habitation sites well-positioned to visually control the sea (TV3; Fig. 3c). When we include locations in areas of high visibility then we can also consider Degrat an early site with good visual control over sea (VNC3; Fig. 4c). Grand Abaque 1 is particularly badly positioned for this purpose. Anse Petite Rivière and Anse à la Gourde are located in areas with average visual control over the sea (TV3; Fig. 3c), although regarding locations embedded in local areas of high visibility then we can also consider Anse Petite Rivière a later site with good visual control over sea (VNC3; Fig. 4c). The sites belonging to the early phase micro-style areas also do not share visibility of certain natural features, such as parts of seascapes and landscapes. The views they offer seem rather to be complementary (CV1–4; Fig. 5a–d). Moreover, smoke columns rising up from villages belonging to the same micro-style area are not intervisible. The fact that strong stylistic similarities do not occur between sites that are particularly close to each other, or that are intervisible, indicates that purposeful efforts were made to maintain close contacts with groups at larger distances. It may be hypothesised that the larger the distance, the larger the need for constructing strong ties between communities.

Interesting to note is that sites related to micro-style areas do not occupy the important positions in networks one would imagine, except for Pointe Colibri and to some extent Anse à la Gourde in the network with only habitation sites (N1; Figs. 7 and 8). We may conclude that visibility was not a prime factor in determining the location and role of key villages like Anse à la Gourde and Anse Petite Rivière. They are by far the largest sites in the micro-region, both belong to a micro-style area and display evidence of materials obtained through short and long distance contacts, yet their visual properties are not exceptional.

### Later Phase Transformation and Defensive Locations

The results show that the two sites on the La Désirade plateau edge dated to the late phase of the Late Ceramic Age, Morne Souffleur and Morne Cybèle-1, are not particularly hidden from view whilst being close to areas from which access routes to these sites, *via* the coast and the sea, can be visually controlled. Both sites are in areas with high local variability in visual properties as revealed by the VNC experiments. They offer particularly limited visual control over the sea but are very close to areas offering great vantage points (TV3; Fig. 3c; VNC4; Fig. 4d). In addition, smoke columns at these sites would be visible from much larger sea areas than those in their immediate surroundings but they are also close to areas where they would be far less visible (TV1; Fig. 3a; VNC2; Fig. 4b). This seems contrary to what one would expect for sites in supposedly defendable locations. In hostile situations, village inhabitants are expected to wish for clear look-out facilities at a site, and to be well-hidden at the same time.

To some extent, other uninhabited areas were better suited to serve a defensive purpose. Many areas in the hilly western and eastern tips of La Désirade and on the plateau edge share the same visual properties of Morne Souffleur and Morne Cybèle-1. Parts of these areas were also occupied during the earlier phase of the Late Ceramic Age, so we cannot argue that the visual properties of Morne Souffleur and Morne Cybèle-1 are exclusively a phenomenon belonging to the later phase. However, since these are the only two sites on La Désirade in this later phase and the experiments reveal that their site locations do serve this purpose, we can assume that these visual features were considered particularly important in this phase and that these locations were purposefully selected partly for their visual properties. The eastern areas of La Désirade might have been avoided because they would not offer great views over the southern coast facing Petite Terre. It is not clear why the western areas were avoided. One reason might be a wish to be not in sight of groups travelling north (for example coming from Marie-Galante or further south) through the passage between Pointe des Châteaux and La Désirade.

A number of earlier sites on La Désirade (*e.g.* Anse Petite Rivière and Pointe Colibri) are better located to visually control seascapes when compared to the later sites of Morne Souffleur and Morne Cybèle-1, from which nevertheless a large area of the sea along the south coast can be visually controlled (CV5–6; Fig. 5e, f; CV10–11; Fig. 6d, e). We can therefore conclude that the ability to visually control the sea from these later sites is not exceptional as compared to earlier sites. However, unlike Anse Petite Rivière and Pointe Colibri, visual properties at Morne Souffleur and Morne Cybèle-1 can change radically within the local area, and the inhabitants of the sites could have profited from precisely this variability. This interpretation again emphasises the importance to understanding visual properties of sites from a landscape perspective and not just from the perspective of the individual sites, as is usually done.

To conclude, it remains questionable whether the locations for Morne Cybèle-1 and Morne Souffleur were selected primarily for their defensive aspects. Villages should preferably be invisible from the sea as smoke columns can easily give away hidden village locations to enemies, whereas good look-outs over the surrounding landscapes and seascapes should be available close-by the village. This is precisely the opposite of what our total viewshed results show (TV1, 3; Fig. 3a, c; Table 4). On the other hand, the visual neighbourhood configuration experiments did reveal that such locations with a defensive nature exist close-by due to the high variability of visual properties in the local area (VNC2, 4; Fig. 4b, d; Table 7), and the cumulative viewshed experiments and the authors’ personal observations during survey work highlight the visibility of the landmasses of Petite Terre and Pointe des Châteaux (CV10–11; Fig. 6d, e). These results reflect a situation that may well have served a defensive purpose, although maybe not exclusively so. If the site locations were not selected for defensive purposes alone, and if they were meant to be seen from a distance, this might also relate to the suggested special, possibly ceremonial, function of the sites, based on the presence of the shell faces or *guaizas* at both sites (De Waal 2003, 2006; Hofman 1995; Hofman et al. 2004). In addition, we could argue that sites participating in regional networks, which both sites were, are not expected to be hidden.

## Conclusions

We started this paper by questioning how visual properties of natural and cultural landscapes in East-Guadeloupe affected human behaviour in pre-colonial times, particularly with regards to interactions between communities, and whether these visual properties and roles changed over time.

We conclude that only few sites (and very few villages) in Eastern Guadeloupe are located in areas that are particularly suitable to visually *control possible sea routes* for short- and long-distance exchange. Even the prominent village of Anse à la Gourde is not in a location that favours visual control of the sea. Other locations in an agglomeration may have provided complementary visual properties and other settlement features allowing villages such as Anse à la Gourde and Anse Petite Rivière to fulfil their roles in exchange networks, for example by functioning as observation outposts.

Contrary to what we had expected on the basis of the presumed importance of the visibility of smoke columns with regards to intersite and interisland contacts, relatively few habitation sites were actually located in high visibility areas. It has been suggested that other elements in the landscape may have helped canoeing people navigate to specific sites or landforms and that neighbouring sites in high visibility areas played a complementary role.

The experiments further suggested that visual control over sea areas and visibility of smoke columns were not factors of importance for the existence of the *micro-style areas*. The stylistic similarities are not related to the visual properties of site locations.

The visibility network experiments have indicated that during the early phase of the Late Ceramic Age networks per landmass are connected and dense and that they incorporate all sites. This structure would allow hypothetical *smoke signalling networks* between sites to allow communication throughout the research area. This probably was a multi-scalar phenomenon: local networks of intervisibility between regularly spaced habitation sites on landmasses connected with more regional long-distance intervisibility of landmasses (Fig. 10).

With regards to *changes that occurred during the later phase of the Late Ceramic Age* and the supposed preference for defensive locations, it can be concluded that the visual properties of the Morne Souffleur and Morne Cybèle-1 site locations were not ideal for defensive purposes, although the inhabitants may nevertheless have profited from the high local variability in visual properties.

In addition to this detailed description and interpretation of the visual properties of the East-Guadeloupe study area, *this paper has made two key contributions* to Caribbean archaeology and landscape archaeology.

First, we used a variety of formal methods to explore how visual properties could have structured past human behaviour. These greatly extend the range of commonly used formal visibility methods in Caribbean archaeology in particular and, when used in combination, enable specific and complex hypotheses to be explored in Caribbean archaeology and landscape archaeology as a whole. We by no means exhausted the range of visibility theories and methods that can be usefully formally explored and argue future research should further extend this range (for example, by exploring the visibility of fire during night-time or navigation using the positions of sun, moon and visible stars). We strongly believe that the more common use of formal approaches already well established in landscape archaeology in general will enable archaeologists to build arguments for the importance of visibility on reproducible and formally comparable results. Importantly, a formal approach will always need to be guided by archaeological theories, contextual knowledge and conducted alongside personal observations. For example, we were highly interested and surprised by results dispelling the importance we as archaeologists attributed to visual properties in a number of cases: the micro-style areas do not seem to be related to visual properties; the major sites of Anse à la Gourde and Anse Petite Rivière do not have the exceptional visual properties one would expect to see for communities that are clearly pivotal in local and regional exchange networks; the visual properties of the late sites of Morne Souffleur and Morne Cybèle-1 cannot be unambiguously interpreted as serving a defensive purpose. In these cases, the formal approach challenged our personal observations and led us to rethink the role of visibility alongside other factors in our archaeological theories. Such studies with negative but useful results, comparing diverse hypotheses and dispelling some of our thoughts about the importance of visibility, are becoming increasingly common in archaeology, a trend we hope will persist (*e.g.* Eve and Crema 2014; Gillings 2015).

Second, the approach presented here recognises that our archaeological theories frequently concern visual properties of landscapes rather than sites. The total viewshed, visual neighbourhood configurations and visibility network methods enable us to formally express such theories, and explore these for site locations as well as the landscape as a whole (to account for our incomplete knowledge of the past settlement pattern). As already concluded earlier (De Waal 2011: 80–81), information on neighbouring sites, archaeological site patterns and elements from the landscape setting clearly enhances the understanding of “landscape use over time and [on] village organisation and interaction among the pre-Columbian Amerindians who once inhabited Pointe des Châteaux, La Désirade and Petite Terre”. We recommend future archaeological visibility studies to avoid a restrictive focus on point site locations and take a landscape perspective enabled by the use of the formal methods used here. For example, the interesting study of seascapes as connecting space by Joshua Torres and Reniel Rodríguez Ramos (2008) could be complemented with total viewshed analyses, whereas studies of the visual properties of sites (*e.g.* Cooper 2010) can be complemented with visual neighbourhood configurations and total viewsheds to evaluate how exceptional sites’ visual properties are as compared to the landscape and sites’ direct surroundings. This approach led us to rethink our archaeological hypotheses explicitly from a landscape perspective rather than a site-centric perspective.

**Fig. 11:**
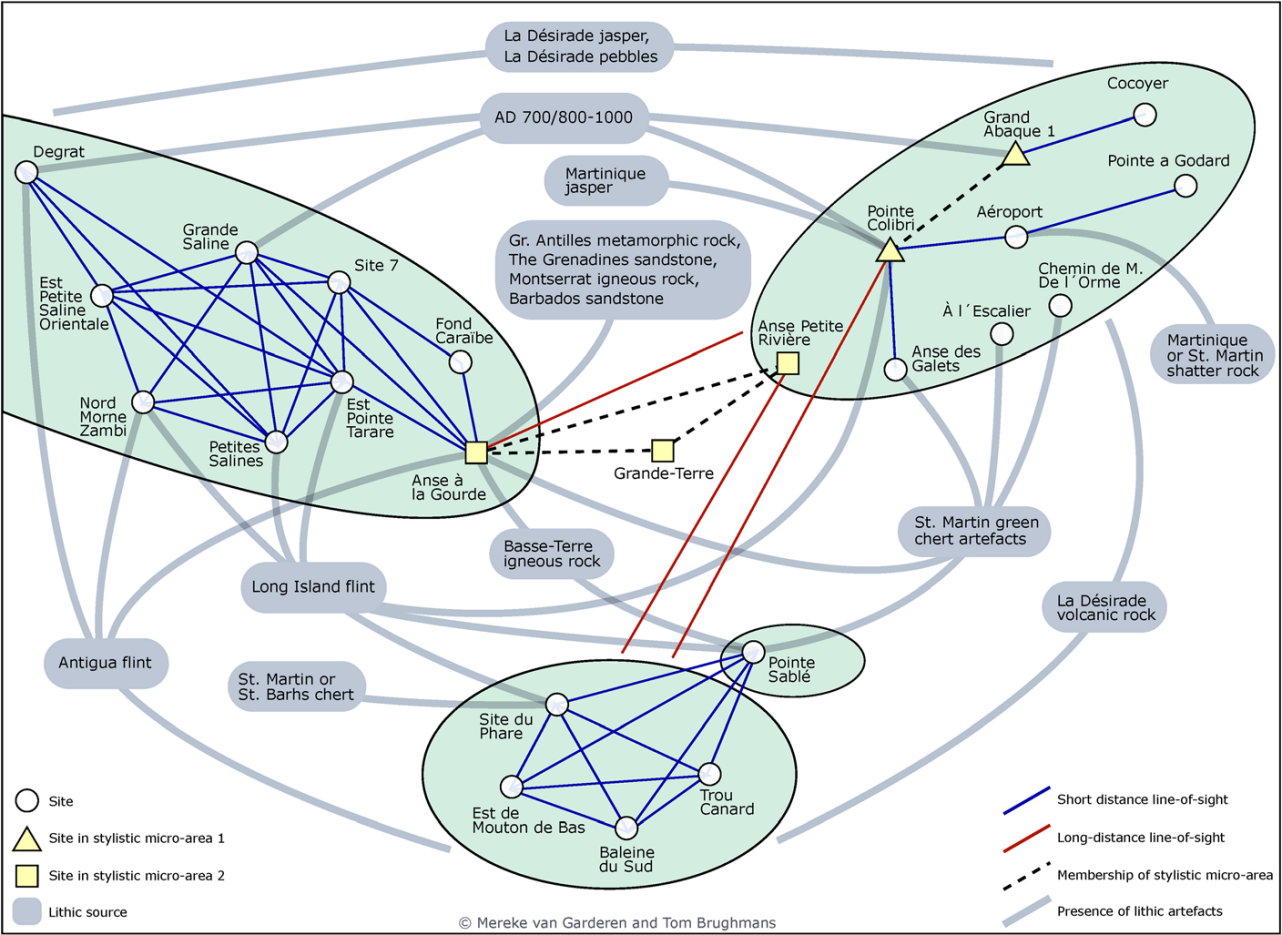
Contextualisation of visibility networks in the East-Guadeloupe study area. Sites are represented as white nodes and grouped per landmass (*green circles*). *Square sites* belong to stylistic micro-area 1; *triangular sites* belong to stylistic micro-area 2. *Grey nodes* represent lithic sources and contemporary dates. *Blue lines* represent short-distance lines-of-sight (N1); *red lines* represent key long-distance line-of-sight (Fig. 10); *grey lines* represent presence of lithic artefacts at sites; *black dashed lines* represent sites’ membership of stylistic micro-areas. Figure created by Mereke van Garderen and Tom Brughmans

A landscape-wide visibility perspective is but one contribution to a more holistic understanding of the multi-scalar interactions of past communities in the Caribbean and should be embedded in further studies addressing different aspects of these interactions. Indeed, in a number of cases mentioned above, the results presented in this paper do not succeed in clarifying artefact similarities and the hypothesised roles of sites, or they simply disprove that visibility had any role to play. Most notable is the case of the major sites of Anse à la Gourde and Anse Petite Rivière that do not have the exceptional visual properties one would expect to see for communities that are clearly pivotal in local and regional exchange networks (Table 3). Future work should therefore continue this line of research that does not consider settlements as bounded entities from a Western perspective, but rather as being integrated into complex sprawling landscape-wide multi-scalar networks. Figure 11 clearly indicates the complexity of these networks, presenting sites within their natural and cultural context and with neighbouring sites in their agglomerations, complementing settlement features. It also depicts sites embedded in local and regional visibility and interaction networks, as evidenced by the presence of non-local materials and close similarities in styles in material culture. The effects of seasonality and palaeo-vegetation, memory and long-term development of local knowledge, and the role of celestial bodies in local and micro-regional visibility studies and navigation between pre-colonial communities remain to be investigated in order to complement the natural landscapes of such networks.

## Appendix. Description of data and methods

### Site data and digital elevation model

The site data include the sites surveyed and studied by Hofman and Hoogland (Hofman 1995) and De Waal (2006) as presented in Table 1 and Fig. 1. This site inventory, including data collected until 2000, presents the most comprehensive documentation of the Late Ceramic Age settlement pattern in East-Guadeloupe. It was created through a systematic survey applying a standardised set of criteria and methods of documentation and analysis for each site, and site information is therefore comparable. An exception is the site of Anse à la Gourde where extensive excavations took place over a period of 7 years as a collaboration between the Service Régional de l’Archéologie de la Guadeloupe and the Faculty of Archaeology at Leiden University resulting in an excavated surface area of more than 2000 m^2^ (Hofman et al. 1999, 2001, 2014). Alongside descriptions of site locations, research history and finds, it records the presence of site location variables that are compared to the visual properties of sites: fresh water, flat terrain, accessible bays with canoe landing spots, reefs, soils suitable for small-scale horticulture, lithic raw materials, viewpoints, strategic elevated spots, salinas and mangrove (Table 1). Site areas with an unknown probable extent were represented as circles with areas estimated by De Waal (2006, 90–91, Table 5.1) and modified to avoid sea areas. Cave sites were given a site area with a 1-m radius (Parc à Jojo, Grotte de Grande Anse, Grotte de Morne Blanc and Morne Frégule at La Désirade). Trou Canard has an unknown site area. For this analysis, it was assumed to have had a minimum area of 70 m^2^, equal to the assumed area of the smallest non-cave site in the dataset.

The raster digital elevation model (DEM) used in this paper is the Litto3D® Guadeloupe topographic LIDAR by © SHOM-IGN (2013), distributed under an Open data licence. The DEM has a horizontal resolution of 1 m and was originally collected between February and March 2010. For a number of the visibility analyses described below, this DEM was coarse-grained at the resolutions described in Table 2 by sampling the DEM elevation values with a grid of points regularly spaced at the desired resolution. The DEM uses the WGS84 world geodetic system and the UTM zone 20 North projection, which were also used for all spatial data presented in this paper.

### Total and cumulative viewsheds

A viewshed of an observation point is the set of target points visible from the observation point. A cumulative viewshed is the sum of the viewsheds from a set of observation locations (Wheatley 1995). A total viewshed is the sum of the viewsheds from every single DEM cell and can be considered to represent the cumulative viewshed from every observation point in the landscape (Llobera 2003). The result is a raster where every cell represents the number of other cells in the study area from which it is visible. In this study, we have generated total viewsheds focused on representing the visual properties under study. They represent visibility from every DEM cell in a hypothesised observation area to every DEM cell in a hypothesised target area (*e.g.* from all sea-based cells to all land-based cells; Table 2, Fig. 2).

Experiments were designed (Table 2, Fig. 2) to reflect the maximum possible visible patterns within the constraints of the hypothesis under study. These reflect good atmospheric conditions, a perfect ability for observers to identify features, and no obstructions caused by vegetation. The latter assumption is known to represent an unrealistic situation, as today large parts of the region are covered in dense and shrubby vegetation and the pre-colonial vegetation is expected to have been higher and more dense (De Waal 2006, 64–65). This issue probably results in an overly positive representation of settlement visibility from the sea. It may also have influenced visual control of seascapes, but if this was an important factor for Amerindian people, they are expected to have cleared areas of vegetation in order to optimise observation conditions. The issue is significantly less problematic as well for the identification of high smoke columns from the sea and (to a lesser extent) on land. The experiments will therefore mainly explore these visual properties. The results of visibility analyses presented here should be treated as an upper limit of what is physically possible. They can be further refined as soon as the availability of palaeo-environmental data allows this.

The following variables were determined for each visibility experiment and are presented in Table 2: observer locations, observer height, target locations, target height and maximum viewing distance. To account for the issue of edge effects in total viewsheds, observer and target locations were sampled at 10-m intervals (the resolution of these experiments, see below) within a buffer of the maximum viewing distance (3 km) around the landmasses that represent the study area (Fig. 1). Observer locations at sea and target locations on land were used for experiments representing visibility from canoes at sea to smoke columns on land. Observer locations on land and target locations at sea are used for experiments representing visibility from land to canoes at sea. For experiments representing human observers, human eye level of 1.6 m was assumed and is considered above average height. For experiments representing visibility of and from canoes at sea, a height of 1 and 1.6 m of a human in a canoe was used. However, results for all experiments revealed only minor differences between these heights, and we therefore present here only results with a height of 1.6 m. This additionally has the advantage of assuming intervisibility or reciprocity of lines-of-sight from human observers with an assumed eye level of 1.6 m. Observations during our visibility surveys in Guadeloupe and Grenada (Brughmans in Hofman 2016) suggest an individual on land and a canoe at sea would be visible up to a maximum distance of 3 km, which is the maximum viewing distance used here.

A review of the visual detection of smoke columns from forest fires suggests a maximum detection radius of 13.4 km under poor atmospheric and observer detection conditions and 20.6 km under good conditions (Rego and Catry 2006). However, smoke columns rising up from kitchen or garden fires in small villages would be far less dense, high and voluminous. Our observations during visibility surveys in Guadeloupe and Grenada suggest such columns are visible up to between 1 and 3 km. Under windy conditions the smoke columns were estimated to be 5 m high, and under less windy conditions straight smoke columns were estimated at 15 m high. Smoke columns were most clearly visible when observed from the sea and when contrasted against the dark green colours of a forested background. In the analyses presented here we assumed domestic smoke columns up to 15 m high that are clearly visible up to 3 km, hence reflecting maximum possible visibility. Since the three landmasses in the study area are located more than 3 km from each other, experiment results were analysed independently for Pointe des Châteaux, La Désirade and Petite Terre.

The results of TV experiments reported for each site are the average values for the entire site area. Results for point locations at the centre of site areas were also obtained and are very similar, but these are not reported here.

To calculate viewsheds, we used the open source *Viewshed Analysis* plugin v0.5.1 in QGIS by Zoran Čučković (2016a, 2016b). It is as fast as comparable algorithms in GRASS and ArcGIS, it provides comparable results to these alternatives, and enables the calculation of visibility networks using the same algorithm. It is therefore preferable for comparing the total viewshed and visibility network results presented here. Earth curvature was taken into account (atmospheric refraction set to 0.13). Since the computation of a total viewshed at 1-m DEM resolutions was prohibitively time consuming for larger landscapes, the DEM was coarse-grained to a 10-m horizontal resolution for all total viewsheds presented here (*e.g.* this reduced the computation time of total viewshed experiment TV1 to *ca.* 36 h for La Désirade only). For all cumulative viewshed experiments, a coarse-grained DEM with a 5-m resolution was used.

In addition, visibility can either be perfect or extremely limited depending on the season and atmospheric conditions. We also know that the current vegetation is not representative for the Late Ceramic Age (*cf.* De Waal 2006, 64–65). Although we acknowledge that formal evaluations of these effects need to be incorporated in formal visibility studies, present-day optimal visibility situations have been taken as a starting point. When more quantitative and qualitative data on changes in the natural setting that may have taken place in the past will become available, the effect on visibility studies can be investigated. Finally, the results of our experiments have been interpreted in contexts of navigation and long- and short-distance contacts between communities. In future work, the experiments should therefore be coupled with seafaring models for the study area, in order to obtain a better understanding of travel over seascapes.

### Visual neighbourhood configurations

Some of the visual properties under study here are not merely visual properties of individual locations but rather concern the way visual properties of individual locations relate to those of other locations in their immediate vicinity: whether sites are located in areas of high or low visibility, and whether sites are located in areas of low visibility with good vantage points in their immediate vicinity. These visual properties can be formally explored by comparing the results of locations in total viewshed experiments TV1 and TV3 with results of surrounding locations, using the *visual neighbourhood configurations* method developed by Brughmans et al. *in preparation*.

Four visual neighbourhood configuration (VNC) experiments were designed using TV1 and TV3 as input (Table 2). VNC1 and 3 represent the average visual property value in an area around each cell and were used to study whether sites are embedded in local areas of high or low visibility. VNC2 and 4 represent the visual prominence of each cell within its local area. This is the difference of the cell’s visual property value with the average value in the area; coined as *visual prominence* by Llobera (2003). The visual prominence was used to study whether site locations are embedded in local areas with visually prominent vantage points nearby. All VNC experiments used circular areas with a radius of 150 m around a focal cell. This radius is just larger than that of the largest site area (Anse à la Gourde) and is therefore considered to represent the area surrounding sites, in which inhabitants were aware of the views offered.

### Visibility networks

The intervisibility of smoke columns at sites was studied as visibility networks (Brughmans and Brandes, 2017), in which nodes represent sites and arcs (directed relationships) represent lines-of-sight of maximum 3-km distance from an observer at one site to a smoke column at another site (Table 2; Fig. 2). Since the observer height (1.6 m) and the maximum height of the observed smoke column (15 m) are not the same, we cannot assume intervisibility or reciprocity (Conolly and Lake 2006, 229–230) and the resulting visibility networks were treated as directed networks. Visibility networks were created with the *Viewshed Analysis* plugin v0.5.1 in QGIS (Čučković 2016a, 2016b) using a 1-m resolution DEM. The visibility network (N1) was created to reflect the site areas, with a set of observation points per site spaced equally at 10-m intervals within the hypothesised site area. The number of lines-of-sight between observation points at a pair of sites is represented as an arc multiplicity attribute.

These visibility networks are represented and analysed in Visone v.2.16 (Visone project team 2016) using a number of network science measures that will be briefly defined here within the context of visibility networks. A *connected component* is a subset of sites that do not have lines-of-sight with sites in other components. The *degree* of a site is the number of lines-of-sight it has, and since these visibility networks are directed, the *indegree* of a site reflects the number of other sites from which smoke columns at this site can be seen, and the *outdegree* of a site reflects the number of smoke columns at other sites that can be observed from this site. The *weighted degree* represents a site’s degree weighted by the number of lines-of-sight connecting locations within site areas of pairs of sites (*i.e.* the degree weighted by the multiplicity attribute of each arc). Finally, the *betweenness centrality* measure was used to explore the hypothesis that this visibility network could have functioned as a smoke signalling network, where communication between sites is made possible through visible smoke signals. A site’s betweenness centrality score reflects how important this site is as an intermediary for passing on information in this signalling network.
